# Helix 8 in chemotactic receptors of the complement system

**DOI:** 10.1371/journal.pcbi.1009994

**Published:** 2022-07-21

**Authors:** Szymon Wisniewski, Paulina Dragan, Anna Makal, Dorota Latek

**Affiliations:** Faculty of Chemistry, University of Warsaw, Warsaw, Poland; University of Maryland School of Pharmacy, UNITED STATES

## Abstract

Host response to infection involves the activation of the complement system leading to the production of anaphylatoxins C3a and C5a. Complement factor C5a exerts its effect through the activation of C5aR1, chemotactic receptor 1, and triggers the G protein-coupled signaling cascade. Orthosteric and allosteric antagonists of C5aR1 are a novel strategy for anti-inflammatory therapies. Here, we discuss recent crystal structures of inactive C5aR1 in terms of an inverted orientation of helix H8, unobserved in other GPCR structures. An analysis of mutual interactions of subunits in the C5aR1—G protein complex has provided new insights into the activation mechanism of this distinct receptor. By comparing two C5aR receptors C5aR1 and C5aR2 we explained differences between their signaling pathways on the molecular level. By means of molecular dynamics we explained why C5aR2 cannot transduce signal through the G protein pathway but instead recruits beta-arrestin. A comparison of microsecond MD trajectories started from active and inactive C5aR1 receptor conformations has provided insights into details of local and global changes in the transmembrane domain induced by interactions with the Gα subunit and explained the impact of inverted H8 on the C5aR1 activation.

## Author summary

Anaphylatoxin C5a produced as a host response to bacterial infections triggers the complement cascade through two chemotactic receptors: C5aR1 and C5aR2. Despite being classified as G protein-coupled receptors, these two receptors transduce the signal in two different ways—one with G protein and the other one with beta-arrestin only. Here, we demonstrated that microsecond molecular dynamics simulations can be used to discriminate one of these signaling pathways for a certain C5aR receptor subtype as indeed nonexistent. This functional selectivity of C5aR receptors results from their structural differences involving not only the receptor core, but also an inverted helix H8 of a much less amphipathic character in the case of C5aR2 than in C5aR1. In addition, transmembrane helix TM6 is shorter in C5aR2 compared to C5aR1, and thus the intracellular region consisting of TM5, ICL3 and TM6 is not stable enough to form any interactions with the G-alpha subunit. Conformational changes in C5aR1 contributing to the beginning of its activation were observed in changing over simulation time contact maps that were generated for interresidual distances and changed over the course of the simulation.

## Introduction

The human immune system responds to SARS-CoV-2 on various levels, out of which a cytokine storm and an organ damaging, pro-coagulant state seem to be the most fatal [1,2]. Both these immune system responses are activated by anaphylatoxins C3a and C5a. These effector molecules attract, activate, and regulate innate and adaptive immune cells [3], leading to the formation of membrane attack complexes (MACs). MAC-associated lysis of bacterial membranes is a major function of the complement system, involving more than 30 soluble and surface-expressed proteins [4]. The complement system comprises C1-C9 convertases, including C3 and C5 cleaved to C3a and C5a, respectively. Three activation pathways of the complement system: classical (C1, C2, C4), lectin (C2, C4) and alternative (C3) converge at the stage of C3 convertase formation and its cleaving of C3 into C3a and C3b leading to the formation of C5 convertase. The final cytolytic MAC is formed by components C5b (from C5), C6, C7 and C8. While MAC disrupts the phospholipid bilayer of pathogens, the degradation products of C3b label cells for phagocytosis, and the C3a and C5a anaphylatoxins evoke the chemotaxis of immune cells.

The activation of C3aR and C5aR receptors by their respective components (C3a and C5a) of the complement system leads to the transmission of proinflammatory signals [4]. Although it is required for the host defense against pathogens, an excessive inflammatory reaction, including the overproduction of cytokines, is also a major cause of tissue damage, e.g., in COVID-19 [5]. Components of the complement system represent druggable targets in the treatment of inflammatory diseases, including rheumatoid arthritis and neuroinflammation [6]. The key to the success of complement system therapies is maintaining the homeostasis of the immune system rather than turning it off to stop the excessive inflammation [7]. Eculizumab, the anti-C5 monoclonal antibody, is one of few currently used drugs targeting the complement system in treatment of blood-brain barrier impairments, e.g., neuromyelitis optica. PMX53 and PMX205 are peptide antagonists targeting C5aR1 in the treatment of neurological disorders [8]. As shown in phase I and II clinical trials, these two cyclic peptides are well-tolerated and more potent than non-peptide W54011 and JJ47 [9], and less violating the Lipinski rule-of-five [10].

Most of the effects of C5a are mediated through the C5aR1 receptor, while the actual role of C5aR2, the second receptor for C5a which was discovered in 2000, is still under investigation. The cellular localization of C5aR2, either surface or intracellular, is also not clearly defined and highly depends on the cell line/type and experimental conditions [11]. Similarly, there are unresolved discrepancies regarding C5aR2 ligands—whether it binds only C5a/C5a des Arg or also: C4a, C3a, and their degradation products C4a des Arg and C3a des Arg (ASP). Many studies describe C5aR2 as a dual-acting receptor, which except for its proinflammatory properties also reduces the inflammatory reaction as a decay receptor, trapping the C5a anaphylatoxin and thus limiting the C5aR1 activation [12]. The regulation of C5aR1 activation [12] also involves carboxypeptidase, which cleaves the C-terminal Arg and converts C5a into its desarginated form (C5a des Arg). Although C5a des Arg also binds to C5aR1 and C5aR2 [13] it is a less potent anaphylatoxin [12] that also triggers other signaling pathways, e.g., HSPC mobilization or lipid metabolism [14].

Functional differences between C5aR1 and C5aR2 have implications in their signaling pathways. C5aR1 transduces the inflammatory signal through the G protein classical pathway while C5aR2 does not interact with G proteins and thus is not considered a conventional GPCR receptor [15]. However, both receptors efficiently recruit β-arrestins [16], which leads to desensitization. C5aR2 has not yet been characterized but known crystal structures of C5aR1 include helix 8 (H8) of the inverted orientation, unobserved in other GPCR structures. Known crystal structures of C5aR1 lack the ICL4 loop and C-terminus, which could explain whether inverted H8 is an artefact or indeed a distinct feature of chemotactic receptors.

Here, we used crystal structures of C5aR1 [17,18] as templates to generate its inactive conformations. Active conformations of C5aR1 and its complexes with G_i_ protein subunits were generated based on FPR2, the most similar template structure available in PDB. We also performed homology modeling to characterize conformations of another subtype of C5aR receptors—C5aR2 and explain its lack of ability to couple with G proteins and affect chemotaxis through the classical G protein-mediated pathway like C5aR1 [16,19]. Based on microsecond MD, we observed that the inverted orientation of amphipathic H8 in C5aR1 did not form any steric hindrance that could prevent interactions of this receptor with G protein subunits. In contrast, H8 in C5aR2 was much less amphipathic and did not form any typical interactions with G protein subunits during simulations, thus confirming its lack of the GPCR-like signaling pathway. In addition, we observed the influence of only the G protein subunits, not involving agonists, on the C5aR1 receptor conformations following Latorraca et al. [20].

## Results

### Validation of crystallographic data for C5aR1

Most of the GPCR structures determined so far include a regular H8 helix of varied lengths. For example, H8 in the GCGR structure (4L6R) is more than 25 residues long, while H8 in endothelin receptor B (6IGL) is only 10 residues long. An intact H8 is essential for the mechanosensation of GPCRs [21] and interactions with signaling proteins [22] and it may contribute to the adjustment of the receptor to alternative signaling pathways [23].

Yet, some GPCR structures include unfolded H8 which is, e.g., involved in receptor-receptor interactions in the crystal lattice (CXCR4 [24]) or no H8 at all (e.g. FFAR1 [25]). Notably, CRFR1 indeed includes a regular H8 helix (shown by cryo-EM in 6P9X [26]) even though its previously determined crystal structures bound to the CP-376395 antagonist (4K5Y and 4Z9G) did not include any H8. Mentioned above unfolded H8 in CXCR4 is questionable if we consider the crystal structure of another close subtype of CXC receptors—CXCR2 (6LFL) which was solved later [27] and included regular H8. For all these reasons we decided to compare known crystal structures of C5aR1, that included inverted H8, regarding the quality of the crystallographic data behind them.

An inverted orientation of H8 is present in all three currently available crystal structures of C5aR1, determined independently by two groups: 5O9H [18], and 6C1Q and 6C1R [17]. We performed an analysis of these three structures using tools available through PDB and the Uppsala EDS server. In all three cases, the information provided in [17,18] concerning the crystallization, structure solution, refinement, and validation was consistent with the data available in PDB.

In the former case (5O9H), the experimental X-ray data presents over 99% completeness up to the highest resolution shell (2.7Å) with reasonable diffracted intensities (I/σ = 7) and a satisfactory Rmerge of 19% given that the data set has been combined from 11 partial sets. The statistics indicates the data is quite reliable. The structural model displays a very reasonable discrepancy factor: R-value of 20.8%, with a very reasonable R-free factor of 23.8%, only slightly higher than R-value. Aside from clashscore and some RSRZ outliers, the other structure validation parameters for this deposition exceed the average data quality expected based on PDB statistics relative to both the whole PDB database and the structural subset of similar experimental data resolution. The clashscore is, however, typical for protein structures of the comparable resolution.

The position and conformation of H8, the direction of which is unusual with respect to other GPCRs, is well supported by the electron density distribution. The 2Fo-Fc map at the sigma contour of 1.5 stands in a very good agreement with the atomic positions of the main chain and supports the presence and conformation of side chains such as P287, L289, V293, L294 and in particular T295 and E296 very well (residue numbering according to PDB). The direction of the carbonyl groups within the main chain of H8 cannot be doubted. The Fo-Fc residual density map at a 1.5 or higher sigma level shows no features in the region of helix-8, indicating a proper model to electron density fit. The electron density of the side chains of solvent-exposed R or E residues is less well-defined. However, these residues are expected to display considerable disorder.

In the case of 6C1Q and 6C1R [17], the experimental X-ray data presents over 86% completeness up to 2.9Å and over 99% completeness up to 2.2Å with reasonable diffracted intensities (I/σ = 6) and very satisfactory Rmerge of 10.6% and 12.8% for 6C1Q and 6C1R accordingly. The latter structure, due to superior completeness and far superior resolution is much more suitable for the discussion of the structural details around the helix H8 fragment, although above statistics indicates the datasets for both structures are equally reliable. The structural model for 6C1R displays a very reasonable discrepancy factor: R-value of 19.2%, with a very reasonable R-free factor of 22.4%, only slightly higher than R-value. Aside from RSRZ outliers, other structure validation parameters for this deposition exceed the average data quality expected based on PDB statistics relative to both the whole PDB database and the structural subset of similar experimental data resolution, suggesting a very reliable structural model. RSRZ outliers are not, however, related to the helix H8 fragment.

The position and conformation of H8 is again well supported by the electron density distribution in 6C1R, even more than in the case of 5O9H. The 2Fo-Fc map at the sigma contour of 1.5 stands in very good agreement with the atomic positions of the main chain. In particular, thanks to the better X-ray data resolution, the orientation of the carbonyl groups within the main chain is clearly indicated. The presence and conformation of side chains such as P402, L404, V408, L409 and, in particular T410, is also very well-supported by the electron density map. The Fo-Fc residual density map at a 1.5 or higher sigma level shows no negative features in the region of H8, indicating a proper model-to-electron density fit. A few positive features indicate that residues N407 and E411 could have been modeled with their side chains (they are replaced by A in the current model, while according to the maps the experimental data clearly contains some information about their side chain-positions). The electron density of the side chains of solvent-exposed R or E residues is less well-defined. However, these residues are again expected to display considerable disorder.

In addition to the above, it is worth noting Sahoo et al. [28], who showed the distributions of the rotameric states of the residues in the molecular switches of C5aR1 in 250 ns MD simulations. This provided more insights into the structural differences between the active and inactive states of C5aR1. Nevertheless, the crystal structures of C5aR1 described here are the only inactive conformations which have been determined so far.

### Differences between C5aR1 and C5aR2 signaling pathways

**Fig 1:**
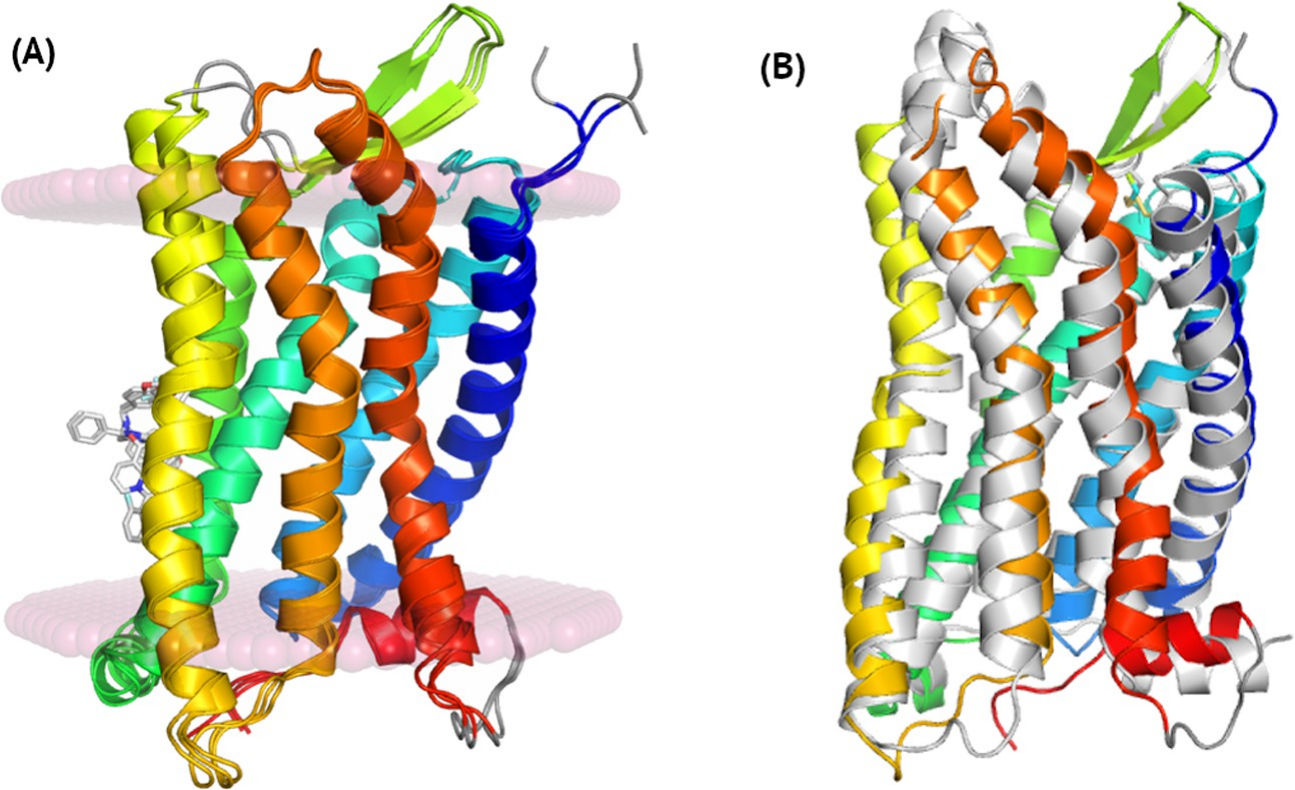
A comparison of the inactive conformations of C5aR1 and an active conformation of FPR2. Crystal structures of C5aR1 shown in (A) represent the inactive conformations of the receptor, bound to inverse agonists NDT9513727 (5O9H), bound to an orthosteric antagonist PMX53 and to an allosteric antagonist NDT9513727 (6C1Q), and bound to an orthosteric antagonist PMX53 and to an allosteric antagonist avacopan (6C1R). Here, only allosteric antagonists NDT9513727 and avacopan were shown and the loops missing in the crystal structures were marked with grey. The inactive conformation of C5aR1 (blue-to-red, 5O9H) shown in (B) was compared with the active conformation of FPR2 (grey, 6OMM). An active conformation of C5aR1 based on FPR2 was included in S1 Appendix and Fig A(I).

The described above crystal structures of inactive C5aR1 lacked a small fragment of ECL2 (an extracellular loop 2), ICL4 (an intracellular loop 4) between TM7 and H8 and C-terminal regions interacting with G protein subunits [16]. These gaps in C5aR1 structures were filled with MODELLER/Rosetta, following procedures described elsewhere [29–31] (see Fig 1A). Then, based on its complete structure, we generated a model of this distinct GPCR receptor in complex with G protein subunits. As a template structure for G protein subunits, we used a crystal structure of a G_i_ protein complex of the formyl peptide receptor 2 (FPR2, PDB id: 6OMM) [32] (see Fig 1B). This receptor was the most similar GPCR receptor to C5aR1 that was deposited in PDB in its active conformation interacting with G protein subunits. However, in these homology models of C5aR1 the receptor was still in its inactive conformation, unadjusted to G protein. For this reason, we also prepared [29–31] a second set of homology models that represented an active conformation of C5aR1, based on the FPR2 template (see Fig 1B).

**Fig 2:**
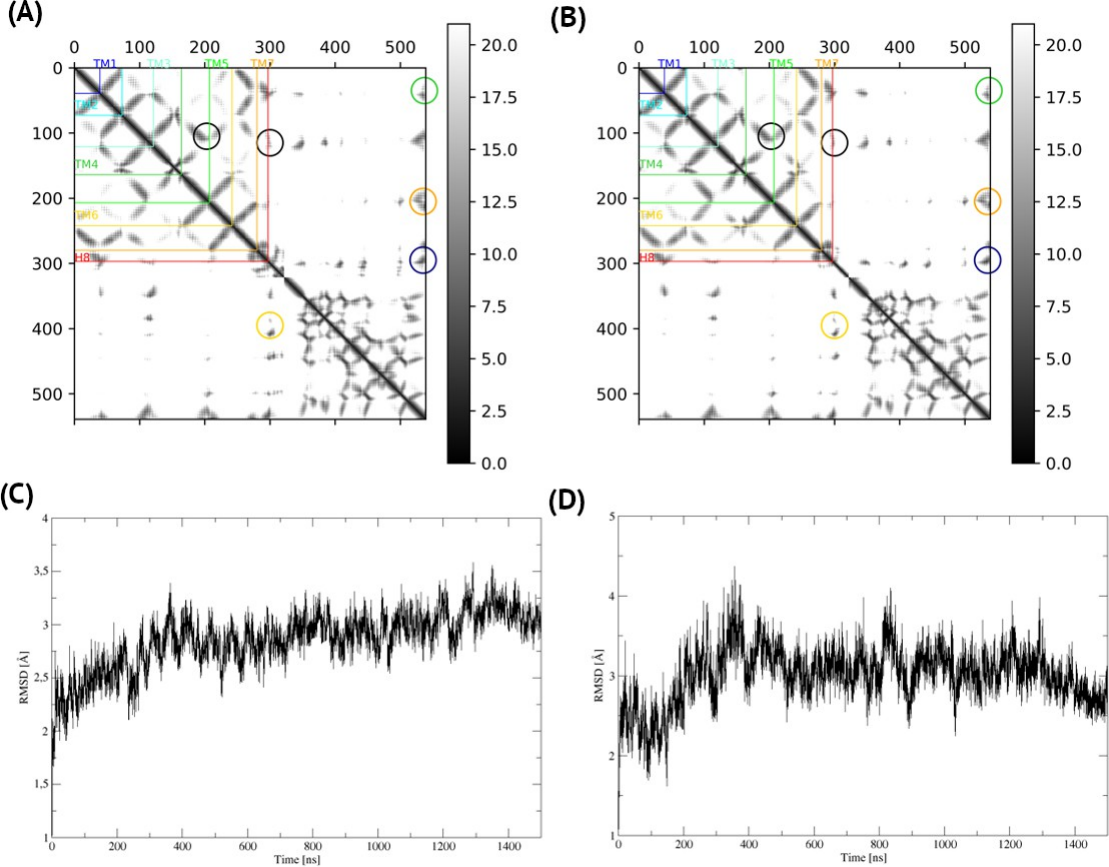
A homology model vs. a microsecond MD-refined model–C5aR1 with G_i_. The homology model of active C5aR1 and G_i_ subunits based on the FPR2 template (A) did not change significantly during microsecond MD simulations (B) which confirmed its reliability. Here, contact maps were shown with the 20 Å cutoff for Cα-Cα distances and the joined, C5aR1 and Gα, sequence numbering. Gβ and Gγ subunits were excluded for the sake of clarity. The C5aR1 –Gα interactions in the C5aR1 C-terminus–Gα–Gβ triple interface (yellow circles) were kept stable (compare A and B). Interactions between C5aR1 and Gα not involving Gβ (green, orange, dark blue circles) also remained the same, similarly to the internal receptor contacts (black circles: left–TM3-TM5 right–ICL2 –C-terminus). The only visible difference was in the receptor sequence region just after H8 (an unstructured, flexible C-terminus) that was adjusted to interact with Gα. A corresponding gradient contact map for the whole simulation trajectory was included as S2 Appendix. (C) The receptor TM core, after a quick adjustment to the lipid bilayer during first 20 ns, remained the same within 3 Å RMSD compared to the starting homology model. The N-terminal part of TM7 and H8 (D) also adjusted during first few ns with the final RMSD not exceeding 3 Å.

**Fig 3:**
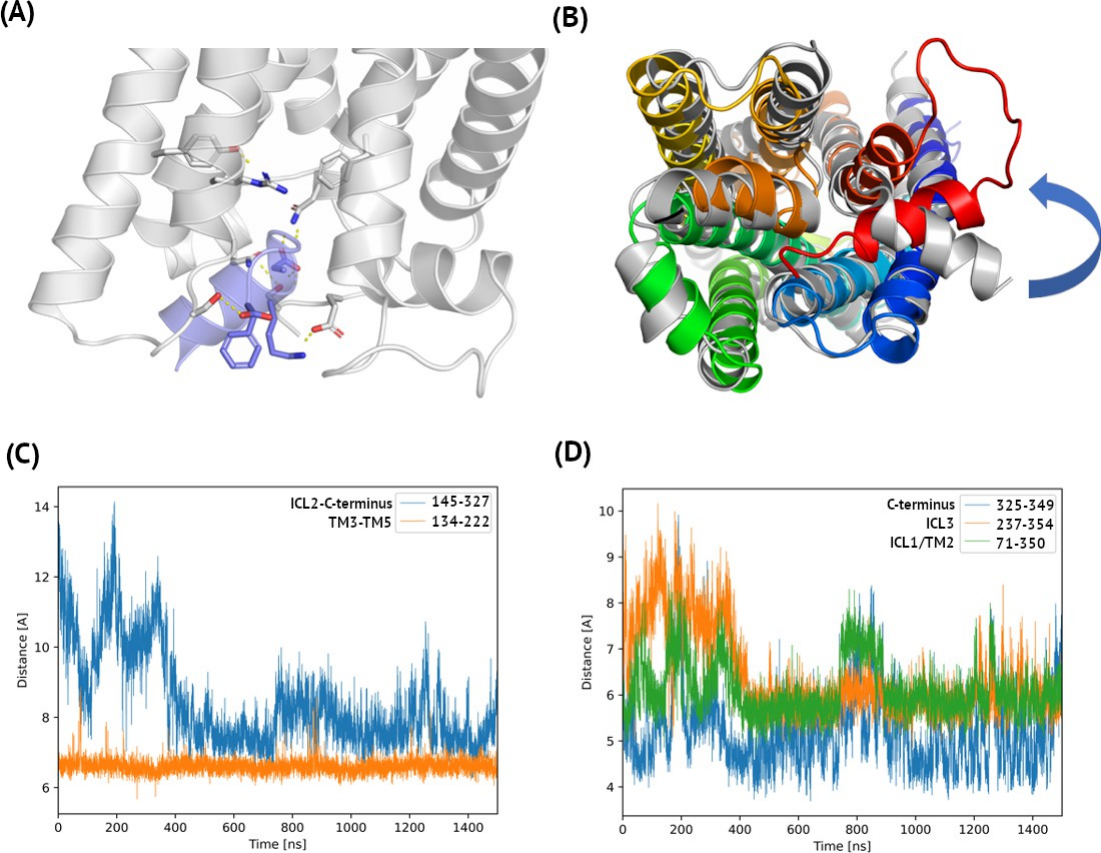
Polar interactions in the complex of C5aR1 and Gα. Here, 1.5 microsecond MD simulations of active C5aR1 was used to analyze the distances between the centers of mass of the amino acids. In (A) a hydrogen bonding network stabilizing the C5aR1 –Gα interface was shown: Asn71 –Asp350, Glu325 –Lys349, Ser237 –Phe354 (C-cap only) in C5aR1 and Gα, respectively. In addition, intrareceptor interactions were shown: Arg134 –Tyr222 and Gln145 –Ser327. These intrareceptor polar interactions stabilized the TM3 –TM5 interface and the ICL2 –C-terminus region. In (B) the C5aR1 –G_i_ complex refined in microsecond MD was superposed on the FPR2 template (6OMM, an intracellular view). The TM core of C5aR1 was shown in blue-to-red (with the C-terminus of Gα in orange) and FPR2 was shown in grey. During simulations, the C-terminal helix of Gα did not change in comparison with the FPR2 template. The C-terminus of H8 in C5aR1 stayed as close to Gα as H8 in FPR2, but its N-terminus was slightly rotated with respect to FPR2. (C) Distance plots for intrareceptor interactions. After ca. 400 ns of the simulation Ser327 (C-terminus of C5aR1) formed interactions with Gln145 (ICL2). The close distance TM3—TM5, represented by the distance between residue 134 (the ‘DRF’ motif—equivalent of ‘DRY’) and 222, was observed during the whole simulation. (D) Distance plots for C5aR1 –Gα interactions. Also in this case, after ca. 400 ns Ser237 (ICL3) formed a stable interaction with C-cap of Phe354 (Gα). Interacting Asn71 (TM2) and Asp350 (Gα) additionally stabilized the complex. The least distance, between Lys349 (Gα) and Glu325 (C5aR1 C-terminus), was kept and did not change during the whole 1.5 μs simulation. Here, sequence numbering was adjusted to P21730 and P63096 entries in Uniprot.

The active conformation of C5aR1 based on FPR2 only slightly changed during microsecond MD simulations (see Figs A and B in S1 Appendix). Fluctuations in TMD remained on the level of 2.5–3.0 Å with respect to the starting homology model of the complex (see Fig 2C). This level of conformational fluctuations refers to the slight changes in loops and side chains of amino acids. Indeed, only a flexible, unstructured ICL4 loop between TM7 and H8 together with EC loops changed their conformations during simulations (see Fig A in S1 Appendix). An intracellular part of TM7 together with ICL4 and H8 was refined during first 200 ns of simulations and kept a stable conformation till the end (see Fig 2D). RMSD values of ca. 3 Å were mostly due to changes in ICL4 and a slight rotation of the N-terminal part of H8 with respect to the template structure (see Fig 3). Notably, the C-terminus of Gα overlapped in both C5aR1 and FPR2 (see Fig 3B). Similarly, H8 in C5aR1 was in the same place, close to Gα, as H8 in FPR2 despite a slight rotation of its N-terminus (see Fig 3B).

**Fig 4:**
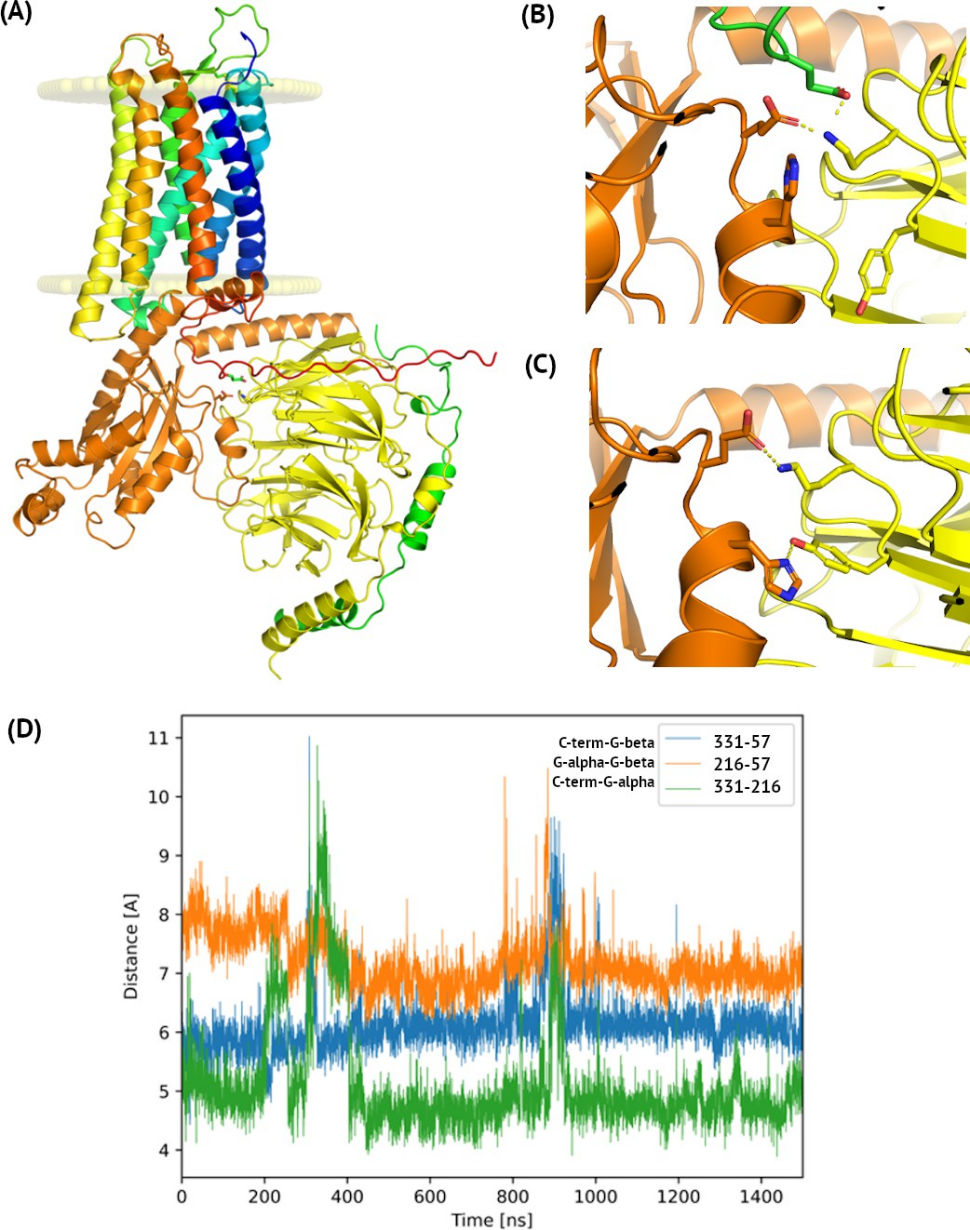
The C5aR1 –Gα–Gβ interface observed in microsecond MD simulations. (A) The full simulation system with the indicated lipid bilayer. Here, the structure of the complex was extracted from the last frame of the microsecond MD simulation started from active C5aR1 based on FPR2 with G_i_. A flexible, unstructured C-terminus of C5aR1 (shown in red) did not form any regular secondary structures during the simulation. (B) Details of the C5aR1 –Gα–Gβ interface. Three polar amino acids were close to each other during the whole simulation: Glu331 (green), Glu216 (orange) and Lys57 (yellow) (C5aR1, Gα, and Gβ, respectively). A similar pattern of interface interactions between Glu (Gα) and Lys (Gβ) residues was observed in the cryo-EM structure of active FPR2 with G_i_ (C). Yet, this structure lacks the receptor C-terminus and instead, only an additional hydrogen bond between His (Gα) and Tyr (Gβ) was observed. (B) A distance plot confirming the three-residues’ interaction in C5aR1. Here, sequence numbering was adjusted to P21730 (C5aR1), P63096 (Gα), and P62873 (Gβ) entries in Uniprot.

Among intramolecular interactions in C5aR1 two hydrogen bonds located in its intracellular part were formed and kept during simulations. These included: Arg134-Tyr222 (TM3-TM5) and Gln145-Ser327 (ICL2-C-terminus) (see Fig 3A and 3C). The former was observed throughout the simulation, while the latter was formed in ca. 400 ns after the mutual adjustment of the complex subunits and was kept till the end (see Fig 3C). On the interface of C5aR1 and Gα, three pairs of amino acids formed stable polar interactions after slight adjustments during first 400 ns. Namely, Asn71 (ICL1/TM2)–Asp350, Glu325 (C-terminus)–Lys349, and Ser237 (ICL3)–Phe354 in C5aR1 and Gα, respectively (see Fig 4C). The last one involved only the C-cap of Phe354. The former two interactions contributed the most to the stabilization of the complex.

During the further analysis of the complex subunits in all simulation replicas, we noticed a three-body interaction including the C-terminal region of C5aR1 (see Fig 4) with Lys57 of Gβ in the center. Three amino acids: Glu331, Glu216, and Lys57 (in C5aR1, Gα, and Gβ, respectively), were close to each other throughout the simulation. Among the three measured interresidue distances, the Glu331-Glu216 distance was the shortest (4.5–5.0 Å, see Fig 4D). A short distance (ca. 4.5 Å) between the centers of mass of Glu331 and Glu216 means that both residues were almost always in a close distance while their negatively charged side chains alternately interacted with positively charged Lys57 (ca. 6 and 7 Å for Glu331 and Glu216, respectively) forming salt bridges. In proteins, two amino acids are in contact if their Cβ atoms are within 8 Å or their Cα within 12 Å [33]. All of the distances fullfilled this requirement throughout the simulation and Glu and Lys are know to frequently form salt bridges [34]. Interestingly, three-body interactions were recently described as essential for an accurate prediction of protein folding rates [35]. The same interaction between Glu and Lys amino acids in Gα and Gβ, respectively, was observed in the template structure of FPR2 (see Fig 4C). Yet additionally, His213 (Gα) interacted with Tyr59 (Gβ). The C-terminus of FPR2 was not present in its cryo-EM structure.

The rest of the inter-subunit interactions in the C5aR1- G protein complex were kept throughout the simulation, which was depicted in Fig 2A and 2B and S2 Appendix including a gradient contact map for the whole simulation trajectory. Except for the C5aR1 –Gα–Gβ interface described above, contacts between C5aR1 and Gα were also kept (see Fig 2A and 2B). What is more, no changes were observed in Gα–Gβ and Gβ–Gγ interfaces. The only noticeble conformational change was a slight inwards movement of the N-terminal helix of Gα (see Fig B in S1 Appendix) closer to C-terminus and H8 of C5aR1. However, Gα–Gβ and Gβ–Gγ interactions were kept throughout the simulation (see S1 Appendix and Fig B).

Global conformational changes during the receptor activation and subsequent disintegration of the complex and further signal transduction via effector adenylate cyclase are hardly accessible even to microsecond all-atom molecular dynamics simulations. Yet, the first steps of the receptor activation can be easily observed in such simulations [36]. Uncovering the driving forces that trigger the signaling cascade provides important clues on how to modify this process with pharmaceuticals. For this reason, we also performed microsecond MD simulations starting from the crystal structure of inactive C5aR1 (see Fig C in S1 and S3 Appendices with a gradient contact map). Contact maps for the whole simulation system included similar patterns of interactions before and after the simulations (see Fig C in S1 Appendix). However, noticeable changes can be observed in contact maps for TM cores only (see Fig C(I-II) in S1 Appendix). Contacts between TM6 and other TMs were changed, confirming the outward movement of its intracellular part upon receptor activation. Indeed, the starting, inactive conformation of C5aR1 based on its crystal structure underwent structural rearrangements leading to the semi-active conformation overlapping with the active conformation of C5aR1 based on FPR2 (see Fig A in S1 Appendix).

**Fig 5:**
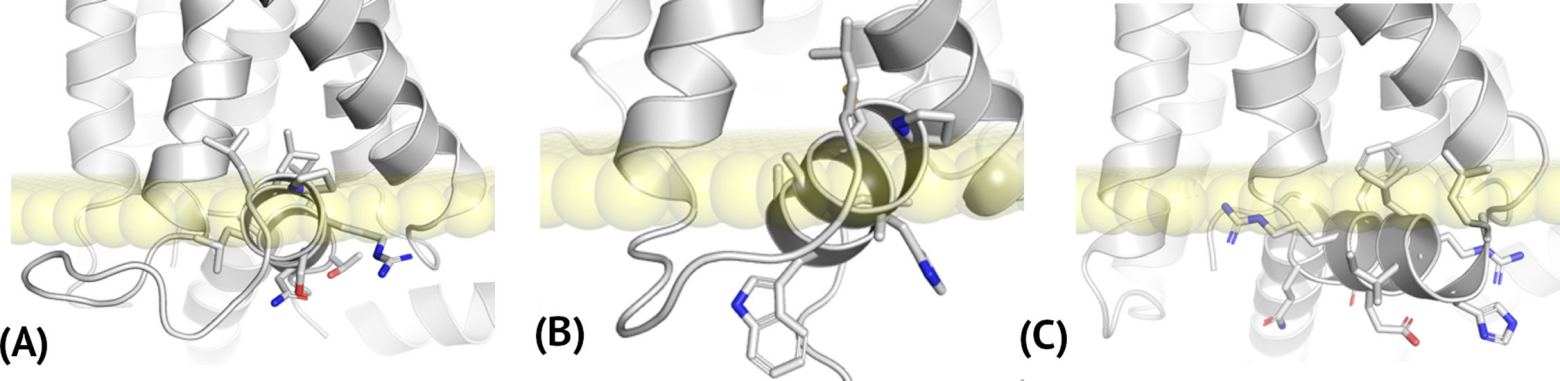
The amino acid composition of H8 in C5aR and FPR2 receptors. (A) C5aR1, (B) C5aR2, (C) FPR1. (A) and (B) structures were extracted from last frames of the microsecond MD simulations of C5aR1 and C5aR2, respectively. Here, homology models of active C5aR receptors based on FPR2 were used to prepare simulation systems. In (A) and (C) interacting Gα subunits were also depicted. C5aR2 (B) was not reported to interact with any G proteins. The location of lipid phosphoryl groups was based on inactive C5aR1 deposited in OPM (6C1Q). The length of H8 in all three cases is similar (ca. 10 residues) but the H8 amino acid composition in C5aR1 and FPR2 differs in comparison to C5aR2. In the case of C5aR1 and FPR2 H8 helices are amphipathic like the typical H8 helices of other GPCRs, but the content of C5aR2 H8, if indeed existing, is mostly hydrophobic on both sites.

In a similar way to C5aR1, though never observed [16], we generated an artificial model of the C5aR2 –G protein complex to prove its lack of stability and any functional implications. Noteworthily, the C-termini in both C5aR1 and C5aR2 lacked any regular secondary structure (see the secondary structure prediction described in Materials and Methods) and demonstrated a high degree of mobility. This flexibility of the C-terminus enables interactions with G proteins and β-arrestin [37]. In a homology model of the C5aR1 –G protein complex (and the artificially generated C5aR2 complex), the C-terminus was in a cleft between the α and β subunits of G_i_ (see Fig 4A). In the MD-refined complex of C5aR2, its C-terminus was not completely relocated from this cleft but it also did not form the same three-residues interactions observed in C5aR1. TM core and H8 of C5aR2 were refined during first 300 ns of simulation and remained stable till the end (see Fig D in S1 Appendix). However, H8 in C5aR2 was moved inside the lipid bilayer during simulations (see Fig 5) because it was less amphipathic than H8 in C5aR1.

**Fig 6:**
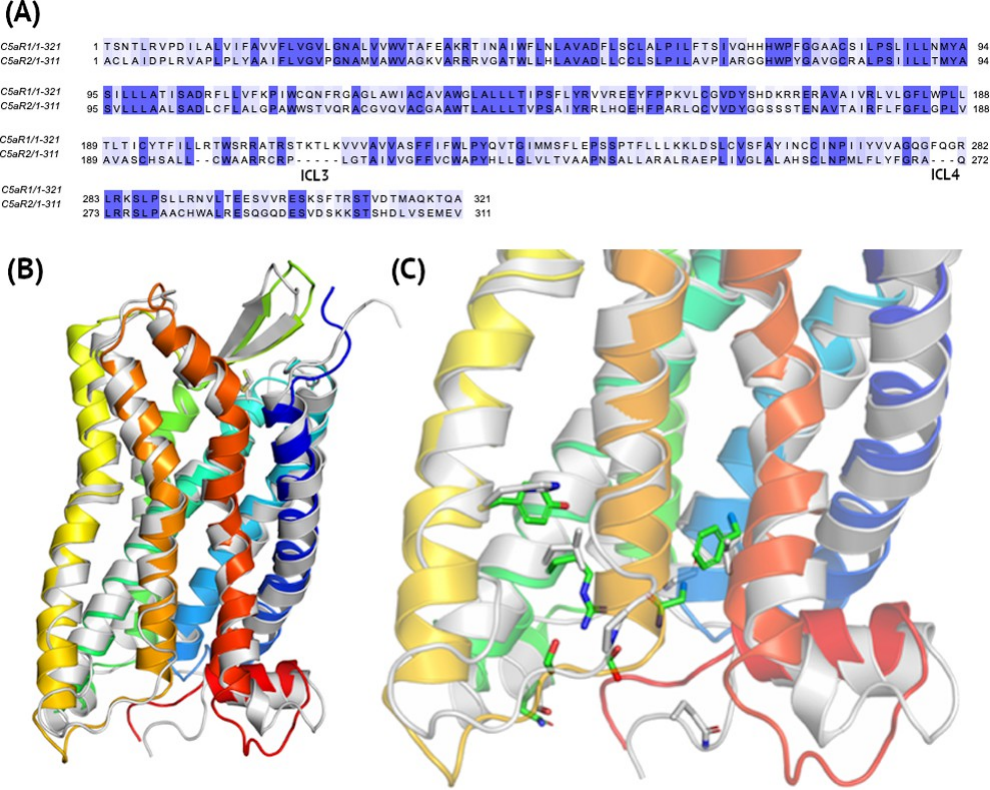
The differences between C5aR1 and C5aR2. (A)–Pairwise sequence alignment with ICL3 and ICL4 loops indicated with 5 and 3-residue deletions in C5aR2. (B) A comparison of the crystal structure of inactive C5aR1 (5O9H, blue-to-red) and a homology model of C5aR2 based on this crystal structure (grey). (D) Amino acid substitutions in the Gα binding region. Here, extracellular 1–29 residues in the N-terminus of C5aR1 and 1–27 residues in the N-terminus of C5aR2 were truncated relative to the sequences deposited in Uniprot (accession numbers P21730 and Q9P296, respectively).

The crucial difference between the TM cores of C5aR1 and C5aR2 was observed in the region of ICL3 (see Fig 6). C5aR2 includes a shorter loop consisting of 7 residues which also implicates its shorter TM6 and TM5 in comparison to C5aR1. Furthermore, ICL4 is shorter in C5aR2 by three residues as compared to C5aR1. However, ICL4 loops in both, C5aR1 and C5aR2, include polar amino acids confirming its outer membrane, intracellular localization (see Fig E in S1 Appendix). Other ECL and ICL loops do not differ to a significant extent (see Fig 6). Yet, Tyr222/His and Arg134/Leu substitutions in C5aR2 disrupt the TM3 –TM5 interactions depicted in Fig 3 for C5aR1. Additional disrupting amino acid substitutions in the Gα binding region included: Asn71/Gly and Ser237/Pro (see Fig 6B). Glu325 was not substituted in C5aR2, yet no interactions with the C-terminal helix of Gα were formed during the simulations (see Fig F in S1 Appendix).

### Helix H8 in C5aR receptors

In both the crystal structures of inactive C5aR1 and homology models of inactive C5aR2 there were no steric clashes between H8 and the transmembrane helices. Also, in the homology models of active C5aR receptors based on FPR2, the inverted orientation of H8 did not overlap with either TMs or Gα. The crystal structure of H8 did not change significantly during MD simulations regardless of the C5aR receptor subtype (see Fig 2D, Fig D(III-IV) in S1 Appendix). This confirms that the inverted orientation of H8 could indeed be a distinct feature of C5aR receptors, not present in other GPCR structures. Nevertheless, the amino acid composition of H8 in C5aR1 was more amphipathic-like than in the case of C5aR2 (see below), which could also explain the differences in the signal transduction of these two receptors.

Amphipathic H8 helices in GPCRs are mainly composed of non-polar amino acids interacting with lipids and polar ones facing the cytosol. For example, in H8 of FPR2, Leu and Phe are located opposite to His, Ser, Glu, Gln and with two Arg on the border (see Fig 5C). The inverted H8 in C5aR1 is composed of three repetitive Leu residues on the lipids side and Arg, Ser, Asn, Thr on the cytosol side with His and two Leu on the border (see Fig 5A). Yet, H8 in C5aR2 only partially follows this amphipathic amino acid composition: two Leu and Cys on the non-polar side and Arg and Trp on the polar one with His, Pro and three Ala residues on the border.

Alanine residues inside the helix and only two polar residues on the intracellular side could be the reason why the N-terminal end of H8 in C5aR2 slightly moved into the lipid bilayer side during simulations (see Fig 5B). This could be the reason why interactions between the receptor and Gα were not established (see Fig F in S1 Appendix). What is more, the shortening of ICL4 in C5aR2 (see Fig E in S1 Appendix) could prevent the proper orientation of H8 with respect to Gα and thus block the Gα-mediated signaling in favor of β-arrestin. Noteworthily, in both C5aR1 and C5aR2, ICL4 consists only of polar amino acids (see Fig E in S1 Appendix), confirming the loop-like conformation of this sequence fragment as not amphipathic, and helical like in case of H8. Therefore, a short helix in ICL4, proposed by the Rosetta cyclic coordinate descent algorithm (see Fig G(II) in S1 Appendix), unfolded during all of the simulations. As was mentioned above, the flexibility of this sequence region was most likely required to properly orient H8 with respect to Gα and TMs (see Fig 3). A helical conformation of ICL4 would stiffen the TM7-ICL4-H8 region and prevent the H8 movement upon the Gα binding during the receptor activation (see below).

As mentioned above, the amino acid composition of H8 helices in C5aR1, C5aR2, and FPR2 reflected their location relative to the lipid bilayer. Typically, amphipathic H8 in FPR2 was located parallel to the lipid bilayer. A similar location was observed for H8 in C5aR1, although it was slightly more immersed into the membrane in comparison to H8 in FPR2. In contrast, H8 in C5aR2 was almost completely covered with lipids with only C-terminal residues facing the cytosol (see Fig 5B). What is more, it was not parallel to the lipid bilayer, but its N-terminus, composed of non-polar residues, was moved slightly closer to the lipids.

Although we do not focus here on the arrestin-mediated signaling of C5aR receptors, we compared their intracellular regions to the corresponding sequence region in β_1_AR binding arrestin-2 (PDB id: 6TKO). As shown in Fig H in S1 Appendix, the arrestin loop in β_1_AR interacts with polar and charged amino acids in ICL1, TM2, TM3 and ICL2. Some of these residues were substituted in C5aR receptors by Ala, which could suggest slightly different arrestin binding sites in these receptors. Interestingly, H8 was in the same place in β_1_AR as in C5aR receptors despite the difference in its direction (Fig H(II) in S1 Appendix), but its C-terminus was not rotated as in case of FPR2 (shown in Fig 3B) but overlayed H8 in C5aR receptors.

### H8 in G protein coupling

As mentioned above, a reliable observation of the GPCR activation requires MD simulations of at least a few microseconds timescale. Yet, our model system remained remarkably stable during all 1.5μs and 1.0μs simulations. The patterns of interactions between C5aR1 and G protein subunits, presented in Fig 2, did not change significantly during microsecond simulations of active C5aR1 conformations. What is more, inactive C5aR1 adjusted its conformation to the G protein subunits during the simulation (see Fig C in S1 Appendix). TM6 moved away from TM7, and TM3 breaking the inactive state TM3-TM6 lock. The C-terminus of C5aR1 and ICL4 adjusted its conformations to fit to the Gα subunit (see Fig C(I-II) in S1 Appendix). Thus, Gα and the other subunits induced conformational changes in the receptor core, as was suggested in [20]. Namely, Latorraca et al. [20] showed that only after G protein binding the population of active state receptors significantly increased. Agonist binding was not enough to change the distribution of the receptor conformations towards the active state. Nevertheless, a much greater simulation timescale is needed to observe further inactive/active state conformational changes. More importantly, simulations starting from an already active conformation of C5aR1 based on FPR2 enabled the observation of a slight rotation of the N-terminus of H8, matching it to Gα (see Fig 3B). This slight rotation of H8 has not yet been observed for simulations starting from inactive C5aR1 as a greater simulation timescale may be required to observe the conformations of the fully activated receptor.

Another distinct feature of the C5aR1—G protein interactions was the three-body interaction (see Fig 4). In the case of C5aR2 this interaction was lost and the C-terminus of C5aR2 moved away from these Gα and Gβ residues (see Fig F in S1 Appendix). This loss of interactions with G_i_ subunits was caused by the lack of any stabilizing interactions between TM6 and H8 of C5aR2, and the C-terminal helix in the Gα-1 subunit (see Fig F in S1 Appendix). Shorter ICL3 and ICL4 loops in C5aR2 compared to C5aR1 have an impact on the length of TM6, which was much shorter and partly replaced by flexible ICL3, which likely prevents the formation of stable interactions with Gα (see Fig 6). Furthermore, a too short ICL4 prevents H8 from adjusting to Gα in the way that we observed for C5aR1 (see Fig F in S1 Appendix). The C-terminus in C5aR receptors is not conserved evolutionary (see Fig 6). A sequence identity on the level of 20% for less than 30 residues sequences is extremely low. This may additionally explain functional differences between these two receptors. Nevertheless, based on our hitherto results, only too short ICL3 and ICL4 loops in C5aR2 indeed prevented its coupling to G protein in a way that no GPCR-like signaling pathway could be observed.

## Discussion

C5aR receptors constitute a distinct example of the evolutionary diversity of sub-fragments in the structures of G protein-coupled receptors. This diversity accounts not only for intra and extracellular loops but also for the amphipathic helix H8. Many crystal or cryo-EM structures of GPCRs includes a disordered H8, e.g., CXCR4 (PDB ID: 3ODU) [38], but none except C5aR1 have included H8 in its inverted orientation so far. Although the disordered conformations of H8 could be explained either by the experimental conditions or the flexibility of this region, a regular but inverted, 10-residues long helix cannot be explained otherwise as it is indeed present in C5aR1.

In this study, we elucidated the details of such a distinct structural motif and its functional implications. Microsecond MD simulations enabled the observation of the first steps of C5aR1 activation and the molecular basis of the G protein coupling. Interestingly, these conformational changes were induced only by interactions with G protein subunits, without the presence of the protein agonist C5a, which followed the suggestion of Latorraca et al. [20] on the impact of G protein binding on changes in the inactive/active state populations during the receptor activation. This forms the basis for further investigations into the full C5a-C5aR1-G protein system involving interactions between C5a and the TMs of C5aR1, resembling the two-sites binding in secretin-like GPCRs as described by Das et al. [39]. Yet, it would require a much larger timescale of simulations than described here to observe such a propagation of conformational changes from the extracellular to intracellular region of the receptor. Nevertheless, first insights on the structural differences between C5aR1 and C5aR2 explaining the functional differences in their signaling pathways were made. Not only were the global rearrangements in the intracellular part of the receptor, e.g., a rotation of the N-terminus of H8 upon Gα binding, but also local conformational changes. Namely, the Glu-Lys-Glu salt bridge in the C5aR1 complex stabilized the three-body interactions between C5aR1, Gα and Gβ subunits. The less amphipathic amino acid composition of H8 in C5aR2, disrupting the parallel position relative to the lipid bilayer, could be one of the reasons why it does not couple with G protein like other GPCRs. This is one of the first examples of the usage of molecular dynamics as a tool to discriminate one of the signaling pathway that is not demonstrated by a certain receptor subtype. The disruption of interactions with G subunits in the case of C5aR2 confirmed its non-GPCR-like signaling pathway. Only C5aR1 properly recognizes G protein subunits and thus can demonstrate the conventional GPCR-like signaling, as was observed in our MD simulations following previous [16] and recent [15] experimental studies in contrast to the arrestin-only signaling of C5aR2. C5aR2 failed to recognize the G protein subunits in MD simulations, possibly because of its less amphipathic H8 compared to C5aR1. Molecular dynamics could be successfully used not only to observe the GPCR signaling on the agonist-receptor interactions level, but also on the level of the molecular recognition of specific signaling proteins by a certain receptor subtype. Although more extensive tests should be performed for other receptors and other types of G protein subunits, it has been confirmed that MD could be used to distinguish between the different signaling pathways of C5aR1 and C5aR2, and to discard signaling pathways that are indeed not observed for certain receptor subtypes.

The rational design of drugs targeting C5aR receptors in the treatment of any inflammatory diseases requires the clarification of their structural and functional differences. The tracking of local interactions crucial to the receptor activation and interactions with subsequent components of its signaling pathway is an inevitable step in understanding these transmembrane proteins of steady interest to pharmacology and medicine. So far, C-terminal regions of these receptors, inaccessible to X-ray or cryo-EM, seem to determine their signaling cascade [40,41], and much more is still to be elucidated with constantly released their new structures [42].

## Materials and methods

### Homology modeling of C5aR receptors

Preliminary models of C5aR1 based on 5O9H, 6C1Q, and 6C1R crystal structures were obtained from GPCRdb [43]. These C5aR1 models were checked for compliance with: Robertson et al. 2018 [18], Liu et al 2018 [17], Pandey et al. 2020 [16]. Most importantly, it was reported there that C5aR2 do not couple with G proteins but interacts with β-arrestin [16]. Pandey et al. [16] proposed that the differences between C5aR1 and C5aR2 regarding the G protein/arrestin signaling pathways were mostly due to this inverted orientation of H8. Previous studies described in [16] suggested that since β-arrestin interacts with both C5aR receptors the loop region between TMH7 and H8 (named ICL4) should be rather of an irregular structure. The N-terminus should not interact with any ligands, while the C-terminus is most likely disordered. This was confirmed by additional secondary structure/disorder regions predictions by RaptorX & Robetta [44,45] (see Fig J in S1 Appendix).

GPCRdb models included a typical orientation of H8 in contrast to PDB structures of C5aR1. The inverted helix H8 has been added based on 6C1Q PDB structures because 5O9H included mutated residues in this region. The missing residues in TMH7 were refined based on the 5O9H structure. The crystal structures of C5aR1 end at R330, similarly to GPCRdb models. But residues that are important for G protein or β-arrestin binding include: Ser334-Thr339, Thr336-Thr342 [16–18]. For this reason, the C-terminus was added in an unstructured, extended conformation by MODELLER.

Based on these preliminary structures of C5aR1 from GPCRdb which were based on: 5O9H, 6C1Q, and 6C1R, we generated full-length models of this receptor using: MODELLER [46], Rosetta [47,48], and GPCRM [29–31]. G_i_ subunits from the template cryo-EM structure of FPR2 (PDB id: 6OMM) were included using MODELLER. In total, 500 TM core models were generated by MODELLER, followed by loop modeling in Rosetta (CCD, 5000 models). 50 top-scoring models were subjected to further analysis. Rosetta models with refined ICL loops, which were missing in the crystal structures of C5aR1 were subjected to short MD simulations (20–100 ns) to select the most stable conformation of this receptor. Two (one helical and the other unstructured) ICL4 loops demonstrated the least RMSD fluctuations and were selected for MD replicas. We selected two versions of ICL4 because of the differences in the results of loop modeling by Rosetta-CCD [49] and Rosetta-KIC [50] (see Fig G in S1 Appendix). The former algorithm favored regular, helical conformations of this loop.

During the next step, the loop models of C5aR1 were inspected in Maestro (Schrodinger, LLC) for protonation/tautomeric states. In principle, during the described above modeling of missing loops, the remaining fragments of the crystal structures were changed as little as possible. Yet, for the following residues rotamers were changed with respect to the crystal structures of C5aR1: Lys185, Thr229, Ser237, Val328, Val329. For example, Thr229 and Ser237 rotamers was adjusted to fit the 5O9H PDB structure (6C1Q and 6C1R lacked the ICL3 loop). As mentioned above, the C5aR1 models were checked for compliance with [16–18] (see Table 1). The residues mentioned in Table 1 were also indicated in Fig I in S1 Appendix, together with the residues mentioned in Figs 3 and 4, for comparison. Especially, the ‘DRF’ motif (referred to as ‘DRY’ in other class A GPCRs) was included in both Table 1 and Fig 3, similarly to the residues involved in the formation of H8 (see Fig I in S1 Appendix).

**Table 1:** The most important residues in C5aR1, based on: [16–18].

Function	Residues in C5aR1^1^
Receptor activation	I124 (I3.40), D133-F135 (‘DRF’ motif)^2^ (G protein coupling), W213 P214 (P5.50), F251 (F6.44), F254-Y258 (‘FWLPY’ motif)^3^, N292, N296-Y300 (‘NPIIY’ motif)^4^ (G protein coupling), S334-T339 (β-arrestin binding), T336-T342 (β-arrestin binding)
Interactions with ligands	F44, L92, I96, P113, L115, N119, I124, A128, T129, F135, I155, V159, A160, L163, L166, L167, R175, R178, V186, C188, E199, R206, L209, W213, P214, L215, L218, I220, C221, F224, F251, Y258, T261, D282, V286
Interactions with Na^+^	N296-Y300 (‘NPIIY’ motif)
Water flow	N55, D82, S114, N119, S171, R175, Y192, R206, T217, W255, Y258, N292, N296
Involved in the H8 formation	L57, V58, V61, F75, Y300, V301, L315, L319, V322, L323

^1^Sequence numbering is the same as in the P21730 entry in Uniprot.

^2^ Refers to ‘DRY’ in other class A GPCRs

^3^ Refers to ‘CWxPY’ in other class A GPCRs

^4^ Refers to ‘NPxxY’ in other class A GPCRs

Complete, curated models of C5aR1 from Maestro were subjected to the HOMOLWAT server [51]. Minor changes were made to remove steric clashes relating to the position of water molecules proposed by this server. The complete models of inactive and active C5aR1 were also used to generate homology models of C5aR2. Both, inactive and active C5aR2 conformations were generated with MODELLER. As a result of the procedure described above, six replicas in total—four replicas for C5aR1 (two for the active and two for the inactive receptor conformations, differing mostly in ICL4 loop conformations) and two replicas for C5aR2 (one for inactive and one for active) were used to generate simulation systems.

### Molecular dynamics simulations of C5aR receptors

Complexes of C5aR receptors were prepared for molecular dynamics simulations using the CHARMM-GUI web server [52] including the conserved ECL2 disulphide bond. Each of the six simulation systems, containing the receptor complex embedded within the lipid bilayer (OPM-oriented [53]) and solvated (TIP3P), was neutralized by the addition of Na^+^ and Cl^-^ ions, with a typical ionic concentration of 0.15 M. The bilayer was formed by POPC and cholesterol molecules with a proportion of 3:1. The number of atoms in each simulation was equal to circa 194000 atoms. The Charmm36 force field was used in each simulation since this force field was tested in various aspects of full-atom simulations of biological systems composed of membrane proteins [54–57]. The equilibration step included six stages, lasting for: 20 ps (steepest descent minimization), 250 ps (conjugated gradients minimization), 250 ps, 250 ps, 500 ps, 500 ps, and 500 ps. During six equilibration stages, the atomic position restraints were gradually released, e.g., for the protein backbone atoms: from 10 (1st stage) to 0.1 kcal·mol−1·Å−2 (6th stage). The first two stages were performed in NVT, the next four in NPT (1 bar, 303.15 K) using the Langevin dynamics. The production run in NPT was performed using the Langevin piston Nose-Hoover method (1 bar, 303.15 K) and lasted more than 1μs (or 1.5μs) for each system. The GPU version of NAMD was used for all MD simulations [58] and every tenth frame was taken for analysis. TM cores quickly adapted to the lipid bilayer during the first 20 ns of production runs. Yet, conformational fluctuations of the whole complex stabilized only after about 300 ns of production runs with the heavy atom backbone RMSD of TMs equal to about 3 Å (C5aR1) 4 Å (C5aR2) and with respect to the starting homology models and did not change after further extension of the simulation time (see Fig 2C and 2D and Fig C(I-II) in S1 Appendix). Noteworthily, a small helix located in ICL4 as proposed by Rosetta CCD (see Fig G(II) in S1 Appendix) was not maintained in all MD simulations even though it was highly populated among Rosetta-generated conformations and assigned the lowest energy due to its regular secondary structure. None of the MD replicas included this helical conformation of ICL4 at the end of the simulation.

## Supporting information

S1 Appendix Figures that were not included in the main text. Fig A. Global changes of C5aR1 induced by G_i_ subunits. Fig B. A homology model vs. a microsecond MD-refined model of active C5aR1 –the location of G protein subunits. Fig C. First steps of the C5aR1 activation observed in microsecond MD simulations. Fig D. Results of microsecond MD simulations performed for C5aR2. Fig E. Amino acid composition of ICL4 loops in C5aR receptors. Fig F. Loss of crucial interactions between C5aR2 and G_i_ subunits. Fig G. A comparison of loop modeling algorithms in Rosetta. Fig H. Beta-arrestin binding site—C5aR receptors vs. β1AR. Fig I. Residues important for C5aR1 function. Fig J. Prediction of secondary structure of C5aR1 and C5aR2.
(PDF)

S2 Appendix A gradient contact map for active C5aR1. Distances were computed between Cα atoms of the receptor and the Gα subunit from every tenth ns of the 1.5 μ MD simulation started from the active conformation of C5aR1 based on FPR2.
(GIF)

S3 Appendix A gradient contact map for inactive C5aR1. Distances were computed between Cα atoms of the receptor from every tenth ns of the 1.5 μ MD simulation started from the crystal structure of the inactive conformation of C5aR1.
(GIF)
