# Supplementary material for: Helix 8 in chemotactic receptors of the complement system
Source: PLoS Comput Biol. 2022 Jul 21;18(7):e1009994. doi: 10.1371/journal.pcbi.1009994 (PMC9359563; doi:10.1371/journal.pcbi.1009994)
Supplement: S1 Appendix — Fig A. Global changes of C5aR1 induced by Gi subunits. Fig B. A homology model vs. a microsecond MD-refined model of active C5aR1 –the location of G protein subunits. Fig C. First steps of the C5aR1 activation observed in microsecond MD simulations. Fig D. Results of microsecond MD simulations performed for C5aR2. Fig E. Amino acid composition of ICL4 loops in C5aR receptors. Fig F. Loss of crucial interactions between C5aR2 and Gi subunits. Fig G. A comparison of loop modeling algorithms in Rosetta. Fig H. Beta-arrestin binding site—C5aR receptors vs. β1AR. Fig I. Residues important for C5aR1 function. Fig J. Prediction of secondary structure of C5aR1 and C5aR2. (PDF) [file pcbi.1009994.s001.pdf]

Appendix S1

Helix 8 in chemotactic receptors of the complement system

Szymon Wisniewski, Paulina Dragan, Anna Makal, Dorota Latek

Faculty of Chemistry, University of Warsaw, Poland

Figure A. Global changes of C5aR1 induced by G_i subunits. (I) - A superposition of inactive C5aR1 (blue-to-red, the C5aR1 crystal structure - 5O9H) and active C5aR1 (grey, the homology model based on 6OMM, the cryo-EM structure of FPR2 with G_i). Both conformations of C5aR1, active and inactive, were subjected to microsecond MD. The structures extracted from the last frames of MD simulations starting from inactive and active C5aR1 were superposed in (II). A global movement of TM6 in inactive C5aR1 towards the active conformation induced by interactions with the Gα subunit was observed. The Gα subunits (orange) also overlapped regardless of the starting model of C5aR1 used for building the simulation system (inactive vs. active).

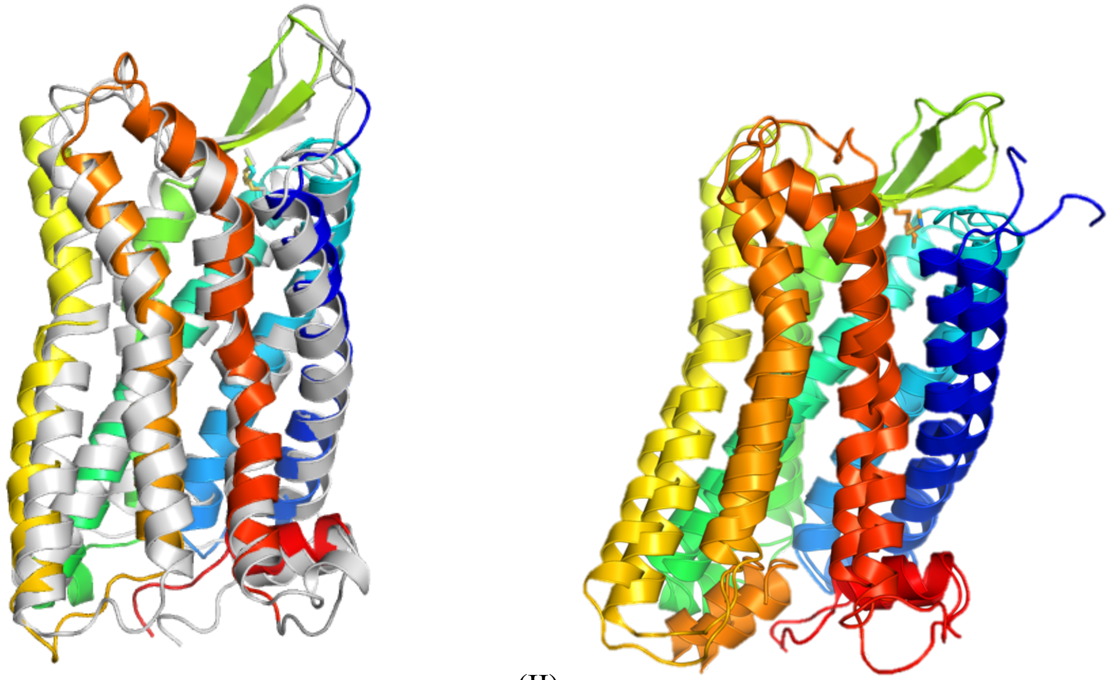

(I) (II)

Figure B. A homology model vs. a microsecond MD-refined model of active C5aR1 – the location of G protein subunits. (I) – An RMSD plot representing the differences in the positions of Cα in the Gα subunit. Here, the TM cores in all of the frames were superposed to show only the variability of the Gα position with respect to the starting homology model. (II) – The same RMSD plot for Gβ. Here, the Gα subunits of each frame were superposed to observe only the

variability in the Gβ position with respect to Gα. (III) – An RMSD plot for Gγ. Here, the Gβ subunits of each frame were superposed to observe the variability in the Gγ position with respect to Gβ. Interfaces Gα – Gβ and Gβ – Gγ were of the least conformational variability. (IV) A superposition of the homology model of C5aR1 based on FPR2 with G_i (dark blue) and the C5aR1 conformation extracted from the last microsecond MD simulation frame (blue-to-red and orange). The N-terminus of Gα was slightly rotated inwards (a green arrow) in comparison to the FPR2 template, but its C-terminus interacting with the receptor remained in the place.

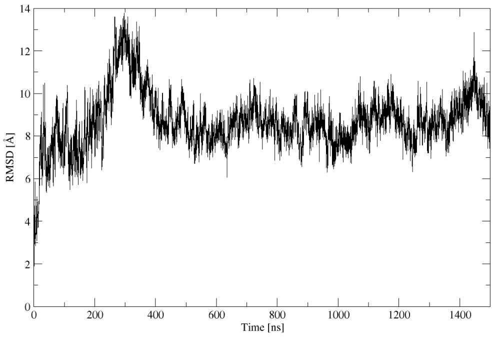

(I)

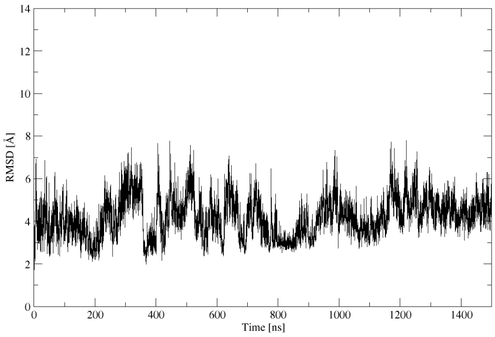

(II)

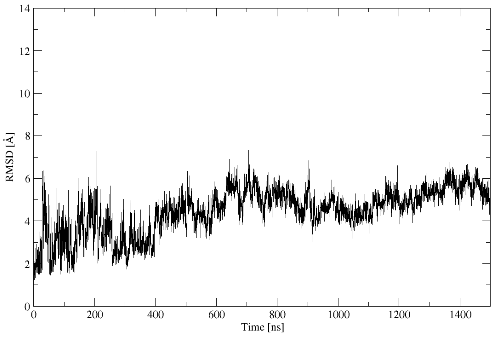

(III)

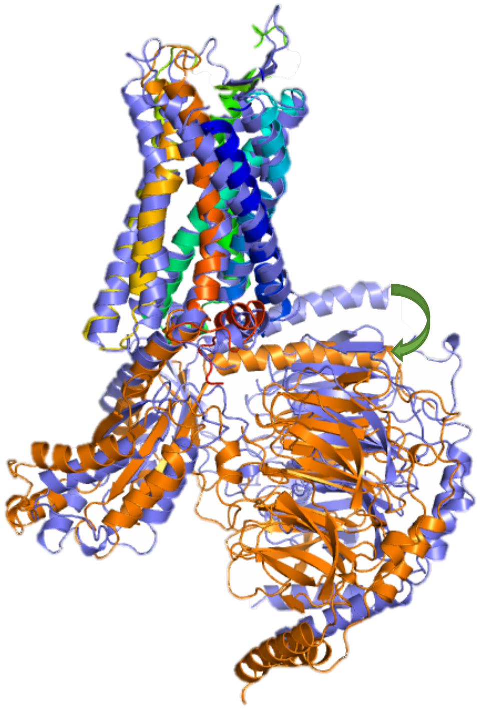

(IV)

Figure C. First steps of the C5aR1 activation observed in microsecond MD simulations. Contact maps generated for a starting homology model of **inactive** C5aR1 with Gα (I, III, V) and for a microsecond MD-refined model of C5aR1 with Gα (II, IV, VI). (I, II) - contact maps for the receptor only. The most visible changes in the receptor conformation were marked with circles (magenta). They included: breaking the TM3-TM6 lock and TM6-TM7 interactions during activation and changes in the TM7-ICL4-H8 region. The most visible was the loss of TM6-TM2 interactions through the TM core of the receptor. (III, IV) - contact maps for the receptor and the Gα subunit. Interacting sequence regions were marked with the same circles as in Fig. 2. In comparison to Fig. 2, a very similar pattern of interactions was obtained at the end of simulations regardless of the starting model - inactive from the C5aR1 crystal structure or active based on FPR2. (V, VI) - contact maps for the whole simulation system. A gradient contact map for the receptor TM core only was included as S3 Appendix.

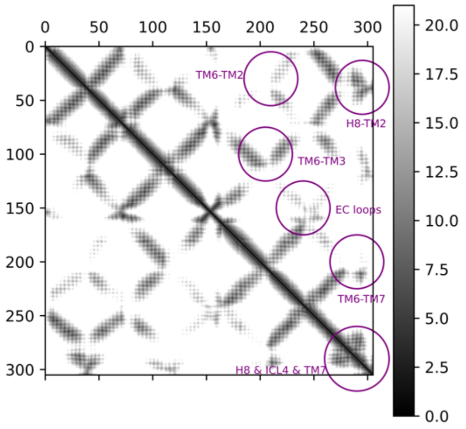

(I)

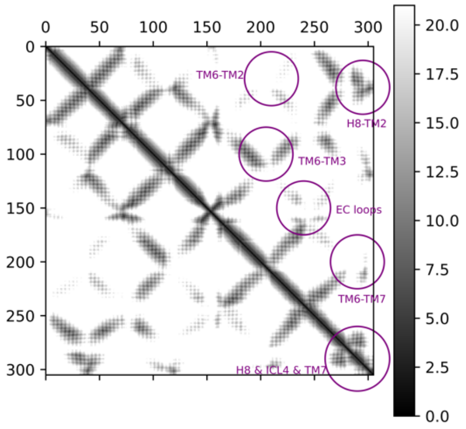

(II)

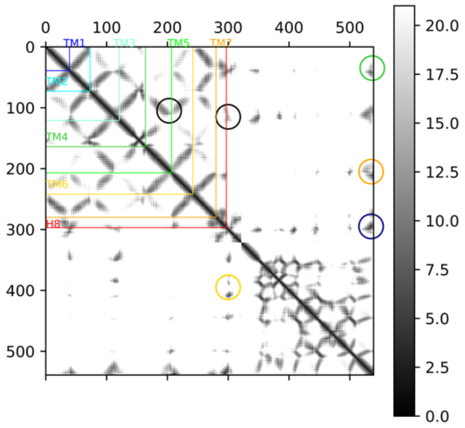

(III)

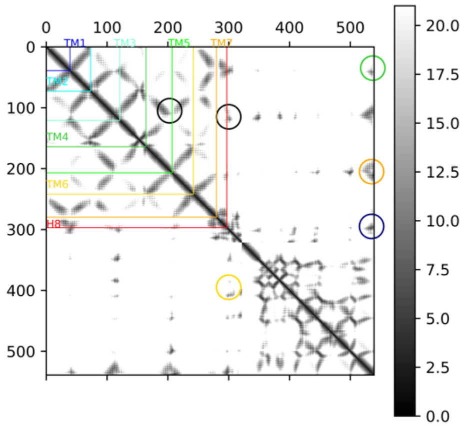

(IV)

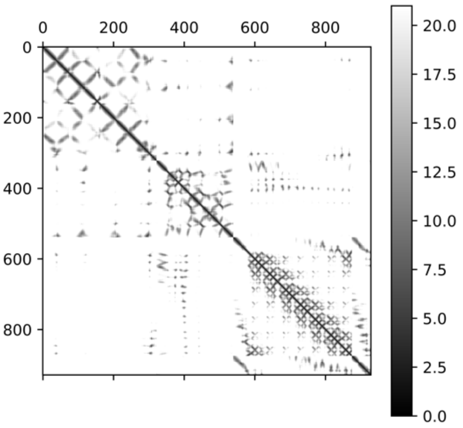

(V)

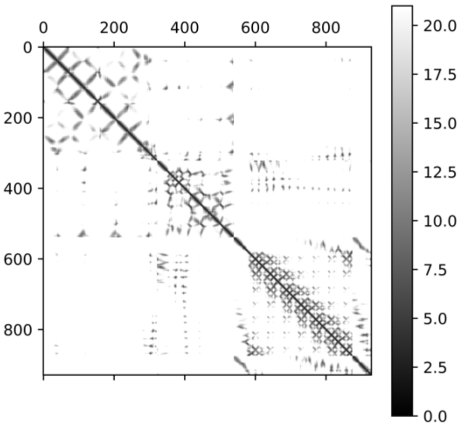

(VI)

Figure D. Results of microsecond MD simulations performed for C5aR2. Here, RMSD was computed for the TM core only, without the flexible C-terminus (I-II), and for the bottom half of TM7 and H8 (III-IV). (I) and (III) correspond to the results of MD performed with the inactive receptor structure based on the crystal structure of C5aR1. (II) and (IV) correspond to the results of MD performed with the active receptor structure based on the cryo-EM structure of FPR2.

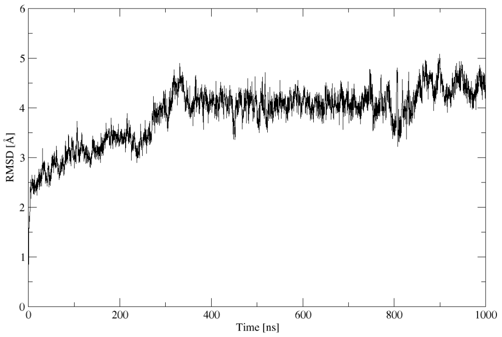

(I)

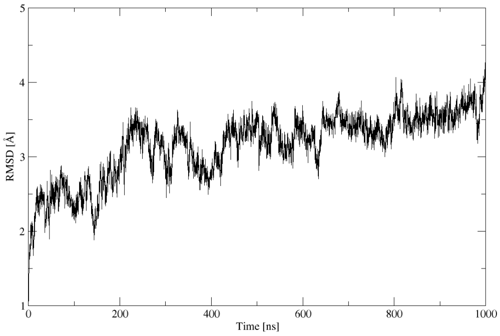

(II)

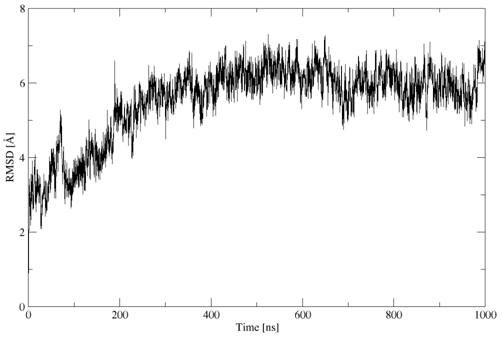

(III)

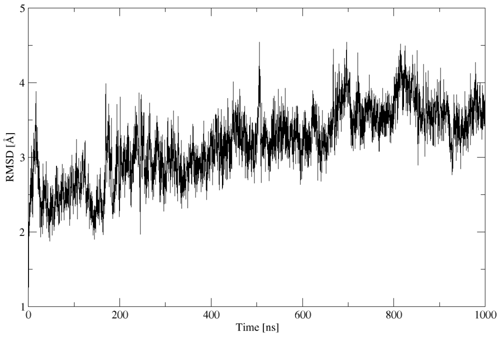

(IV)

Figure E. Amino acid composition of ICL4 loops in C5aR receptors. In both cases, C5aR1 (I) and C5aR2 (II), polar amino acids are the most populated except for one Leu residue, but ICL4 (green) in C5aR2 is shorter than in C5aR1. Flexible ICL4 loops in both receptors did not form any regular secondary structure during MD simulations.

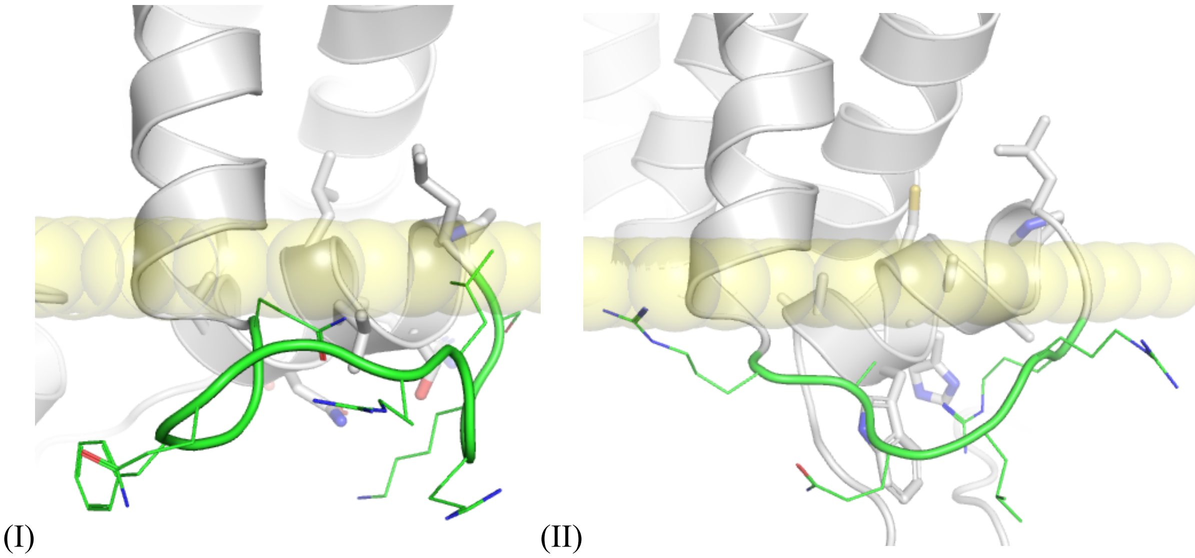

(I) (II)

Figure F. Loss of crucial interactions between C5aR2 and G_i subunits. (I, II) – RMSD plots showing a loss of interactions between C5aR2 and the Gα and Gβ subunits. In both cases, the distances exceeded 8 Å, the maximal distance between the centers of mass of two residues to describe them as still being in contact. (I) – The RMSD plot for the Cα - Cα distance in the C5aR2 complex corresponding to the 325-349 distance in the C5aR1 complex (see Fig. 3D). This shows the loss of interactions between C5aR2 and Gα. (II) - The RMSD plot for the distance in the C5aR2 complex corresponding to the 331-57 distance in the C5aR1 complex (see Fig. 4D). This shows the loss of interactions between C5aR2 and Gβ. (III, IV) - A comparison of the last simulation frames of C5aR1 (grey and dark blue) and C5aR2 (blue-to-red and orange) showed completely different relative positions of H8 and the C-terminus of Gα in these two receptors despite their similar starting conformation based on active FPR2 with G_i. It was caused by shorter ICL3 and ICL4 loops in C5aR2 in comparison to C5aR1. (III) – a side view, (IV) – an intracellular view. Similar loss of contacts between the receptor and Gα can be observed in contact maps (V-VIII). (V, VI) – contact maps generated for the simulation started from active C5aR2 based on FPR2, (VII-VIII) – contact maps generated for the simulation started from inactive C5aR2 based on the crystal structure of C5aR1. Left panels (V and VIII) correspond to the beginning of simulations while right panels correspond to last frames of simulations. Regardless the starting conformation of C5aR2 used for the simulation, loss of several interactions was observed confirming the decreased stability of such simulation system. For example, intra-receptor interactions (ICL2 - C-terminus incl. H8, marked with right black circles) were lost. Also, C-terminal regions of both, the receptor and Gα (dark blue circles) did not interact by the end of simulations so close like in case of C5aR1 (see Fig. 2). Glu325 – Lys349 contact on the C5aR1 – Gα interface (see Fig. 3) was lost in the case of C5aR2. Moreover, there were discrepancies between simulations started from active C5aR2 and inactive one in this region (compare VI and VIII). Finally, regions involved in three-body interactions in C5aR1 (see Fig. 4, yellow circles), in C5aR2 were involved in far fewer contacts (yellow circles) suggesting that such interactions were not present at all.

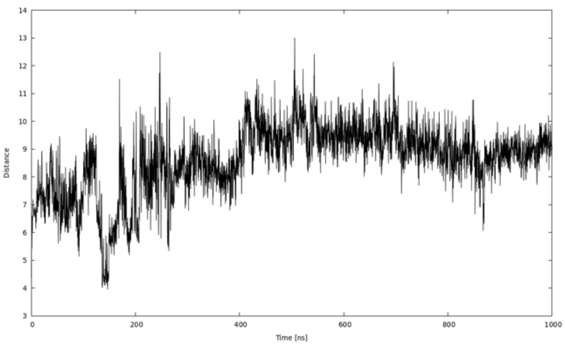

(I)

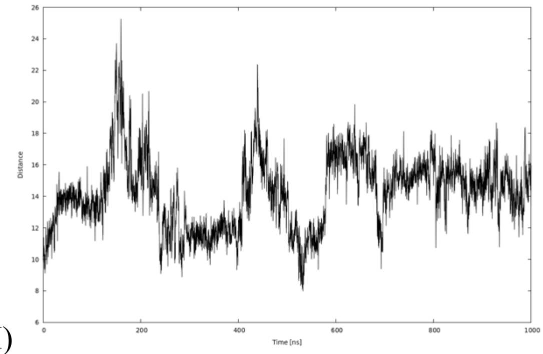

(II)

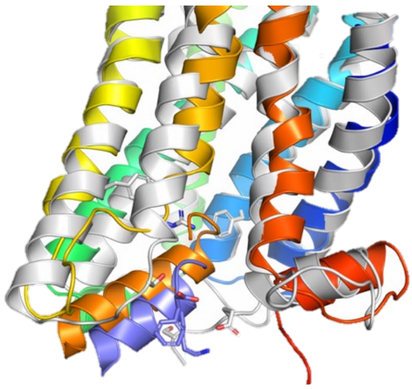

(III)

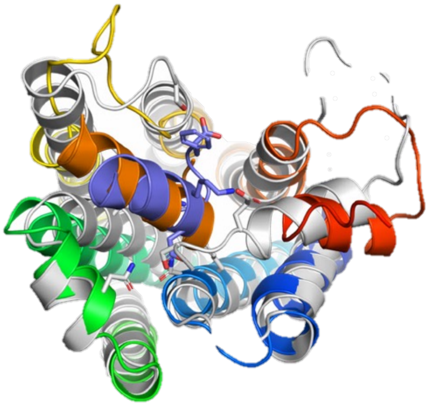

(IV)

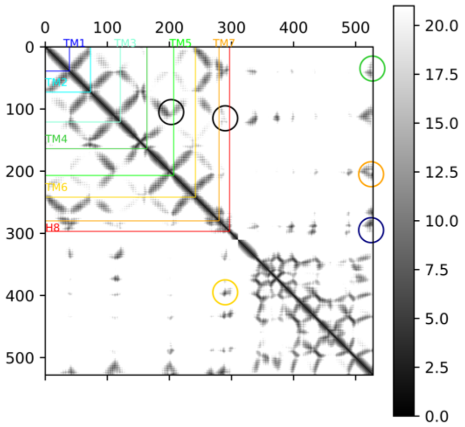

(V)

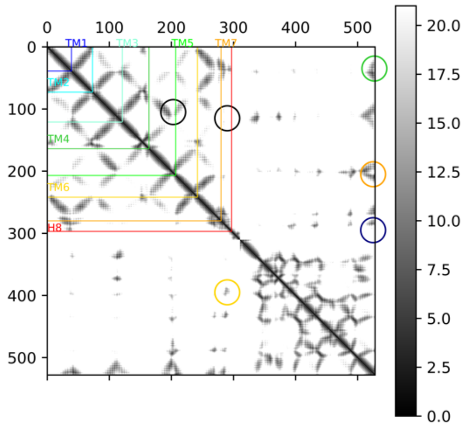

(VI)

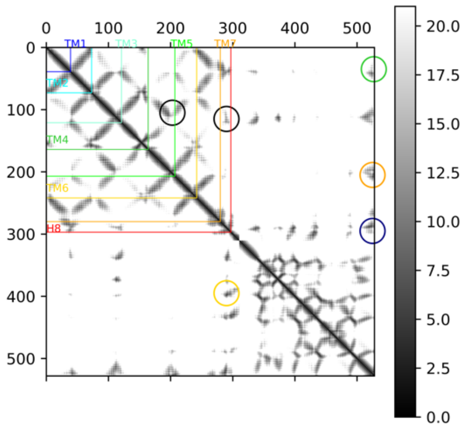

(VII)

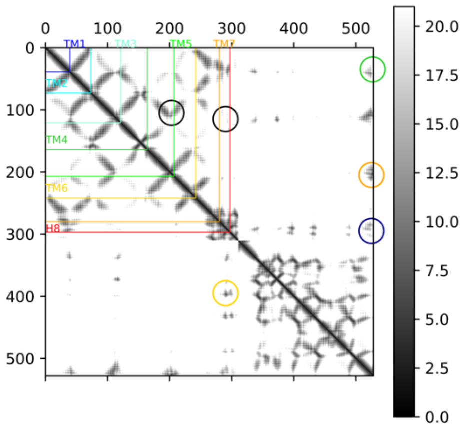

(VIII)

Figure G. A comparison of loop modeling algorithms in Rosetta. KIC (I), CCD (II), 50 top-scoring loop models were selected from 5000 generated models. Here, an inactive structure of C5aR1 (PDB id: 5O9H) was used for loop modeling. CCD allowed for a more extensive conformational search also including TM helices but was slightly biased towards regular secondary structures.

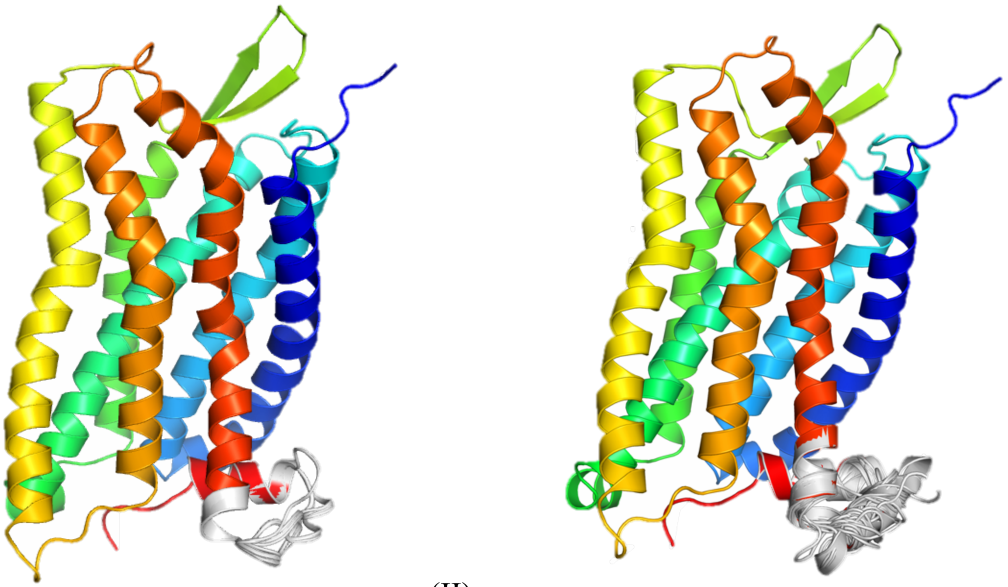

(I) (II)

Figure H. Beta-arrestin binding site - C5aR receptors vs. β_1AR. (I) β_1AR in complex with arrestin-2 (PDB id: 6TKO). Polar contacts were marked with dashed yellow lines. (II) a superposition of β_1AR (green), C5aR1 (orange), and C5aR2 (grey). Here, a crystal structure of inactive C5aR1 and a homology model of inactive C5aR2 based on it were used. For both C5aR receptors, alanine (ICL1 - blue, ICL2 - green) substitutions of active site residues were observed in comparison to β_1AR. Only for C5aR2 Arg/Leu (TM3 – marine green) and Gln/Gly (TM2 – light blue) substitutions were observed. H8 (red) was in the same place in all three receptors (II). Arrestin binding sites in details: β_1AR (III), C5aR1 (based on β_1AR) (IV), and C5aR2 (based on β_1AR) (V). In (II-V) receptors were shown in blue-to-red color schemes.

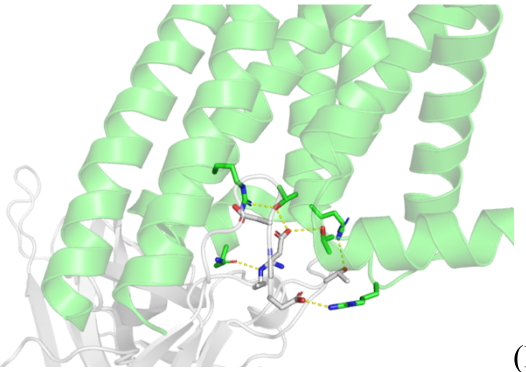

(I)

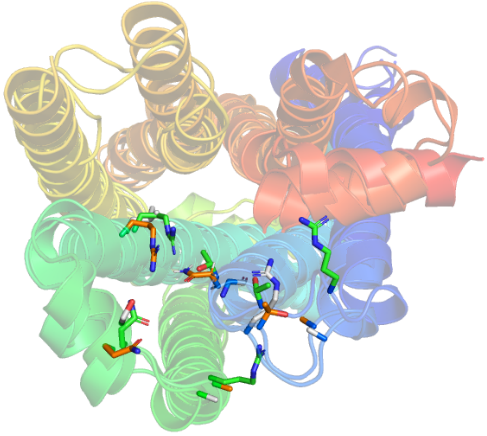

(II)

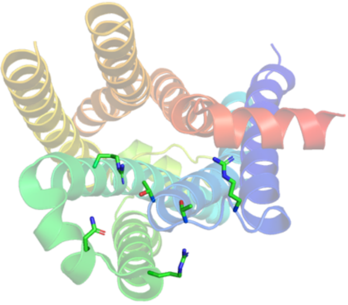

(III)

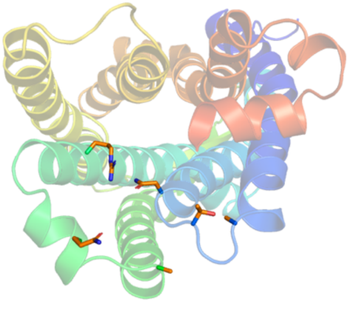

(IV)

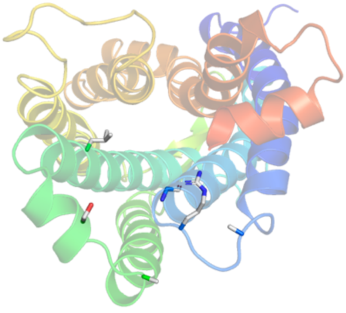

(V)

Figure I. Residues important for C5aR1 function. Here (I - II), the residues described in Figures 3 and 4, involved in the G protein coupling and signal transduction, were shown in orange, with the 'DRF' motif shown in red. The residues described in Table 1 as involved in the receptor activation were shown in yellow, the residues involved in the β-arrestin binding were shown in green, and the residues involved in the formation of H8 were shown in magenta. The latter were shown in detail in (II) – the side view. Both, (I) and (II), represent the refined C5aR1 structure extracted from the last frame of microsecond simulations, started from active C5aR1 based on FPR2.

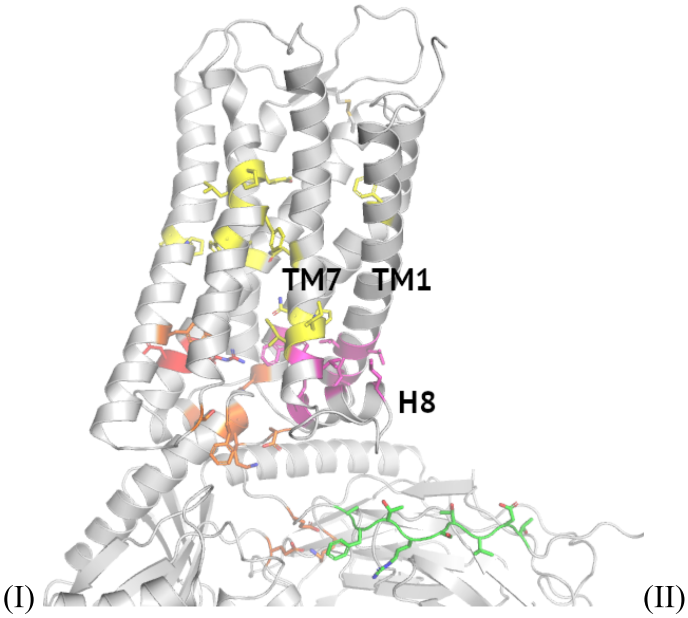

(I)

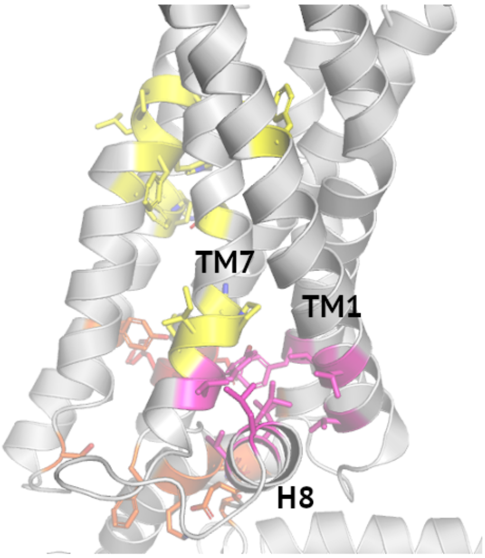

(II)

Figure J. Prediction of the secondary structure of C5aR1 and C5aR2.

```
#DeepConCNF_SS8: eight-class secondary structure predicti
results
#probabilities are in the order of H G I E B T S L(loops)
the 8 secondary structure types used in DSSP
   1 M L   0.000 0.000 0.000 0.000 0.000 0.000 0.000 1.00
   2 D L   0.001 0.001 0.000 0.001 0.001 0.002 0.000 0.99
   3 S L   0.002 0.004 0.000 0.002 0.001 0.014 0.012 0.96
   4 F L   0.003 0.008 0.000 0.002 0.001 0.010 0.012 0.96
   5 N L   0.003 0.007 0.000 0.002 0.001 0.007 0.011 0.96
   6 Y L   0.003 0.007 0.000 0.005 0.004 0.021 0.022 0.93
   7 T L   0.001 0.005 0.000 0.005 0.004 0.025 0.041 0.91
   8 T L   0.001 0.004 0.000 0.003 0.003 0.008 0.088 0.89
   9 P L   0.003 0.009 0.000 0.002 0.002 0.020 0.046 0.91
  10 D L   0.004 0.018 0.000 0.002 0.003 0.018 0.027 0.92
  11 Y L   0.010 0.051 0.000 0.002 0.003 0.043 0.042 0.84
  12 G L   0.011 0.069 0.000 0.001 0.002 0.091 0.051 0.77
  13 H L   0.015 0.081 0.000 0.002 0.003 0.092 0.071 0.73
  14 Y L   0.022 0.068 0.000 0.005 0.006 0.088 0.052 0.75
  15 D L   0.025 0.058 0.000 0.008 0.010 0.073 0.034 0.79
  16 D L   0.024 0.052 0.000 0.005 0.006 0.068 0.071 0.77
  17 K L   0.032 0.088 0.000 0.002 0.004 0.143 0.057 0.67
  18 D L   0.030 0.101 0.000 0.001 0.002 0.157 0.052 0.65
  19 T L   0.033 0.105 0.000 0.005 0.004 0.170 0.044 0.63
  20 L L   0.028 0.047 0.000 0.016 0.035 0.067 0.086 0.72
  21 D L   0.005 0.011 0.000 0.012 0.015 0.006 0.024 0.92
  22 L T   0.004 0.009 0.000 0.003 0.004 0.601 0.044 0.33
  23 N T   0.003 0.007 0.000 0.001 0.002 0.674 0.087 0.22
  24 T S   0.002 0.004 0.000 0.006 0.009 0.003 0.684 0.29
  25 P L   0.015 0.007 0.000 0.016 0.019 0.041 0.041 0.86
  26 V L   0.027 0.031 0.000 0.027 0.060 0.092 0.109 0.65
  27 D L   0.029 0.032 0.000 0.018 0.015 0.085 0.106 0.71
  28 K L   0.035 0.040 0.000 0.006 0.007 0.173 0.154 0.58
  29 T L   0.020 0.027 0.000 0.003 0.004 0.129 0.109 0.70
  30 S L   0.018 0.034 0.000 0.003 0.006 0.073 0.083 0.78
  31 N L   0.021 0.033 0.000 0.007 0.006 0.143 0.116 0.67
  32 T L   0.026 0.024 0.000 0.019 0.025 0.079 0.195 0.63
  33 L L   0.040 0.010 0.000 0.018 0.024 0.014 0.155 0.73
  34 R L   0.067 0.004 0.000 0.009 0.012 0.008 0.024 0.87
  35 V H   0.852 0.006 0.000 0.001 0.000 0.017 0.013 0.11
  36 P H   0.910 0.005 0.000 0.001 0.000 0.016 0.008 0.06
  37 D H   0.968 0.006 0.000 0.001 0.000 0.007 0.005 0.01
  38 I H   0.989 0.001 0.000 0.000 0.000 0.004 0.001 0.00
  39 L H   0.995 0.001 0.000 0.000 0.000 0.002 0.001 0.00
  40 A H   0.997 0.000 0.000 0.000 0.000 0.002 0.000 0.00
  41 L H   0.996 0.000 0.000 0.000 0.000 0.002 0.000 0.00
  42 V H   0.997 0.000 0.000 0.000 0.000 0.001 0.000 0.00
  43 I H   0.997 0.000 0.000 0.000 0.000 0.002 0.000 0.00
  44 F H   0.997 0.000 0.000 0.000 0.000 0.002 0.000 0.00
  45 A H   0.997 0.000 0.000 0.000 0.000 0.002 0.000 0.00
  46 V H   0.996 0.000 0.000 0.000 0.000 0.002 0.000 0.00
  47 V H   0.996 0.000 0.000 0.000 0.000 0.002 0.000 0.00
  48 F H   0.995 0.001 0.000 0.000 0.000 0.003 0.000 0.00
  49 L H   0.993 0.001 0.000 0.000 0.000 0.004 0.001 0.00
  50 V H   0.990 0.001 0.000 0.000 0.000 0.006 0.001 0.00
  51 G H   0.989 0.001 0.000 0.000 0.000 0.007 0.001 0.00
  52 V H   0.991 0.001 0.000 0.000 0.000 0.005 0.001 0.00
  53 L H   0.992 0.001 0.000 0.000 0.000 0.004 0.000 0.00
  54 G H   0.993 0.001 0.000 0.000 0.000 0.004 0.000 0.00
  55 N H   0.994 0.001 0.000 0.000 0.000 0.003 0.000 0.00
  56 A H   0.995 0.001 0.000 0.000 0.000 0.003 0.000 0.00
  57 L H   0.993 0.001 0.000 0.000 0.000 0.004 0.000 0.00
  58 V H   0.992 0.001 0.000 0.000 0.000 0.005 0.000 0.00
  59 V H   0.995 0.001 0.000 0.000 0.000 0.003 0.000 0.00
  60 W H   0.994 0.002 0.000 0.000 0.000 0.002 0.000 0.00
  61 V H   0.985 0.004 0.000 0.001 0.000 0.006 0.001 0.00
  62 T H   0.958 0.007 0.000 0.001 0.000 0.026 0.002 0.00
  63 A H   0.759 0.009 0.000 0.001 0.002 0.148 0.028 0.05
  64 F H   0.326 0.017 0.000 0.003 0.009 0.196 0.260 0.18
  65 E L   0.103 0.034 0.000 0.004 0.010 0.191 0.161 0.49
  66 A T   0.025 0.052 0.000 0.001 0.003 0.582 0.177 0.16
  67 K T   0.011 0.045 0.000 0.002 0.005 0.539 0.098 0.30
  68 R L   0.014 0.029 0.000 0.003 0.008 0.107 0.164 0.67
  69 T L   0.045 0.011 0.000 0.003 0.012 0.029 0.208 0.69
  70 I H   0.855 0.015 0.000 0.001 0.001 0.025 0.030 0.07
  71 N H   0.892 0.011 0.000 0.001 0.000 0.019 0.013 0.06
  72 A H   0.958 0.010 0.000 0.001 0.001 0.007 0.006 0.01
  73 I H   0.990 0.001 0.000 0.001 0.000 0.002 0.001 0.00
  74 W H   0.995 0.001 0.000 0.001 0.000 0.002 0.001 0.00
  75 F H   0.995 0.001 0.000 0.000 0.000 0.002 0.000 0.00
  76 L H   0.996 0.001 0.000 0.000 0.000 0.002 0.000 0.00
  77 N H   0.997 0.000 0.000 0.000 0.000 0.002 0.000 0.00
  78 L H   0.997 0.000 0.000 0.000 0.000 0.002 0.000 0.00
  79 A H   0.996 0.000 0.000 0.000 0.000 0.002 0.000 0.00
  80 V H   0.995 0.001 0.000 0.000 0.000 0.003 0.000 0.00
  81 A H   0.995 0.001 0.000 0.000 0.000 0.003 0.000 0.00
  82 D H   0.992 0.001 0.000 0.000 0.000 0.005 0.001 0.00
  83 F H   0.993 0.001 0.000 0.000 0.000 0.004 0.000 0.00
  84 L H   0.991 0.002 0.000 0.000 0.000 0.005 0.001 0.00
  85 S H   0.985 0.003 0.001 0.001 0.000 0.009 0.001 0.00
  86 C H   0.946 0.006 0.001 0.001 0.000 0.044 0.002 0.00
  87 L H   0.608 0.008 0.001 0.000 0.000 0.371 0.007 0.00
  88 A H   0.665 0.010 0.001 0.000 0.001 0.300 0.006 0.01
  89 L H   0.965 0.003 0.001 0.000 0.001 0.016 0.005 0.00
  90 P H   0.986 0.003 0.000 0.000 0.000 0.009 0.001 0.00
  91 I H   0.991 0.003 0.000 0.000 0.000 0.005 0.000 0.00
  92 L H   0.993 0.003 0.000 0.000 0.000 0.002 0.000 0.00
  93 F H   0.991 0.003 0.000 0.000 0.000 0.003 0.000 0.00
  94 T H   0.988 0.004 0.000 0.000 0.000 0.006 0.000 0.00
  95 S H   0.973 0.005 0.000 0.000 0.000 0.018 0.001 0.00
  96 I H   0.957 0.007 0.000 0.000 0.000 0.030 0.003 0.00
  97 V H   0.902 0.013 0.000 0.001 0.001 0.069 0.008 0.00
  98 Q H   0.739 0.016 0.000 0.001 0.001 0.191 0.024 0.02
  99 H H   0.472 0.020 0.001 0.004 0.008 0.280 0.069 0.14
 100 H T   0.070 0.014 0.000 0.003 0.005 0.438 0.328 0.14
 101 H L   0.019 0.016 0.000 0.010 0.016 0.384 0.168 0.38
 102 W L   0.025 0.021 0.000 0.016 0.026 0.089 0.301 0.52
 103 P L   0.099 0.046 0.000 0.012 0.016 0.294 0.107 0.42
 104 F T   0.265 0.055 0.000 0.008 0.015 0.298 0.076 0.28
 105 G H   0.508 0.048 0.001 0.003 0.008 0.154 0.063 0.21
 106 G H   0.690 0.050 0.001 0.001 0.002 0.170 0.042 0.04
 107 A H   0.724 0.058 0.001 0.002 0.005 0.130 0.027 0.05
 108 A H   0.762 0.087 0.001 0.004 0.008 0.087 0.028 0.02
 109 C H   0.736 0.100 0.001 0.003 0.004 0.119 0.019 0.01
 110 S H   0.639 0.091 0.001 0.000 0.001 0.232 0.015 0.02
 111 I H   0.806 0.043 0.001 0.000 0.001 0.128 0.011 0.01
 112 L H   0.977 0.004 0.000 0.000 0.001 0.009 0.004 0.00
 113 P H   0.989 0.002 0.000 0.000 0.000 0.005 0.001 0.00
 114 S H   0.992 0.001 0.000 0.000 0.000 0.005 0.001 0.00
 115 L H   0.996 0.001 0.000 0.000 0.000 0.002 0.000 0.00
 116 I H   0.996 0.000 0.000 0.000 0.000 0.002 0.000 0.00
 117 L H   0.996 0.000 0.000 0.000 0.000 0.003 0.000 0.00
 118 L H   0.997 0.000 0.000 0.000 0.000 0.002 0.000 0.00
 119 N H   0.997 0.000 0.000 0.000 0.000 0.002 0.000 0.00
 120 M H   0.997 0.000 0.000 0.000 0.000 0.002 0.000 0.00
 121 Y H   0.997 0.000 0.000 0.000 0.000 0.002 0.000 0.00
 122 A H   0.997 0.000 0.000 0.000 0.000 0.002 0.000 0.00
 123 S H   0.996 0.000 0.000 0.000 0.000 0.002 0.000 0.00
 124 I H   0.997 0.000 0.000 0.000 0.000 0.002 0.000 0.00
 125 L H   0.997 0.000 0.000 0.000 0.000 0.002 0.000 0.00
 126 L H   0.996 0.000 0.000 0.000 0.000 0.002 0.000 0.00
 127 L H   0.996 0.000 0.000 0.000 0.000 0.002 0.000 0.00
 128 A H   0.996 0.001 0.000 0.000 0.000 0.002 0.000 0.00
 129 T H   0.996 0.001 0.000 0.000 0.000 0.002 0.000 0.00
 130 I H   0.995 0.001 0.000 0.000 0.000 0.003 0.000 0.00
 131 S H   0.994 0.001 0.000 0.000 0.000 0.003 0.000 0.00
 132 A H   0.990 0.002 0.000 0.000 0.000 0.006 0.001 0.00
 133 D H   0.980 0.003 0.000 0.000 0.000 0.012 0.001 0.00
 134 R H   0.967 0.005 0.000 0.000 0.000 0.021 0.002 0.00
 135 F H   0.949 0.007 0.000 0.000 0.000 0.035 0.002 0.00
 136 L H   0.939 0.009 0.000 0.001 0.000 0.042 0.004 0.00
 137 L H   0.887 0.012 0.000 0.000 0.000 0.093 0.004 0.00
 138 V H   0.827 0.010 0.000 0.001 0.000 0.152 0.006 0.00
 139 F H   0.794 0.007 0.000 0.003 0.003 0.144 0.035 0.01
 140 K L   0.228 0.012 0.000 0.003 0.005 0.093 0.171 0.48
 141 P L   0.264 0.067 0.000 0.005 0.010 0.122 0.056 0.47
 142 I H   0.300 0.084 0.000 0.004 0.009 0.158 0.212 0.23
 143 W L   0.269 0.081 0.000 0.007 0.010 0.133 0.168 0.33
 144 C L   0.287 0.075 0.000 0.004 0.008 0.079 0.152 0.39
 145 Q S   0.291 0.093 0.000 0.002 0.005 0.147 0.320 0.14
 146 N S   0.278 0.100 0.000 0.004 0.005 0.172 0.314 0.12
 147 F H   0.298 0.082 0.000 0.005 0.008 0.157 0.214 0.23
 148 R L   0.292 0.022 0.000 0.009 0.013 0.078 0.147 0.43
 149 G L   0.349 0.009 0.000 0.008 0.015 0.016 0.067 0.53
 150 A H   0.912 0.011 0.000 0.000 0.000 0.032 0.007 0.03
 151 G H   0.945 0.010 0.000 0.000 0.000 0.029 0.005 0.01
 152 L H   0.974 0.009 0.000 0.000 0.000 0.008 0.002 0.00
 153 A H   0.991 0.002 0.000 0.000 0.000 0.003 0.001 0.00
 154 W H   0.995 0.001 0.000 0.000 0.000 0.002 0.000 0.00
 155 I H   0.997 0.000 0.000 0.000 0.000 0.001 0.000 0.00
 156 A H   0.997 0.000 0.000 0.000 0.000 0.001 0.000 0.00
 157 C H   0.998 0.000 0.000 0.000 0.000 0.001 0.000 0.00
 158 A H   0.997 0.000 0.000 0.000 0.000 0.002 0.000 0.00
 159 V H   0.997 0.000 0.000 0.000 0.000 0.002 0.000 0.00
 160 A H   0.997 0.000 0.000 0.000 0.000 0.002 0.000 0.00
 161 W H   0.997 0.000 0.000 0.000 0.000 0.002 0.000 0.00
 162 G H   0.996 0.000 0.000 0.000 0.000 0.002 0.000 0.00
 163 L H   0.997 0.000 0.000 0.000 0.000 0.002 0.000 0.00
 164 A H   0.995 0.001 0.000 0.000 0.000 0.003 0.000 0.00
 165 L H   0.995 0.001 0.000 0.000 0.000 0.002 0.000 0.00
 166 L H   0.990 0.003 0.000 0.000 0.000 0.006 0.000 0.00
 167 L H   0.864 0.004 0.000 0.000 0.000 0.128 0.002 0.00
 168 T H   0.829 0.007 0.000 0.000 0.000 0.152 0.006 0.00
 169 I H   0.853 0.017 0.001 0.001 0.001 0.035 0.039 0.05
 170 P H   0.887 0.058 0.000 0.001 0.000 0.024 0.007 0.02
 171 S H   0.893 0.075 0.000 0.002 0.000 0.019 0.004 0.00
 172 F H   0.886 0.075 0.000 0.006 0.000 0.018 0.007 0.00
```

173	L	H	0.828	0.059	0.000	0.018	0.001	0.058	0.013	0.02
174	Y	H	0.711	0.052	0.000	0.051	0.003	0.129	0.014	0.03
175	R	H	0.492	0.045	0.000	0.083	0.003	0.292	0.025	0.06
176	V	H	0.379	0.035	0.000	0.157	0.010	0.283	0.026	0.11
177	V	L	0.243	0.018	0.000	0.243	0.020	0.070	0.072	0.33
178	R	L	0.176	0.021	0.000	0.264	0.015	0.072	0.058	0.39
179	E	L	0.130	0.057	0.000	0.233	0.017	0.120	0.087	0.35
180	E	T	0.074	0.069	0.000	0.165	0.009	0.244	0.230	0.20
181	Y	S	0.039	0.054	0.000	0.127	0.012	0.258	0.263	0.24
182	F	L	0.009	0.008	0.000	0.072	0.015	0.075	0.279	0.54
183	P	L	0.004	0.009	0.000	0.023	0.007	0.193	0.164	0.60
184	P	T	0.003	0.015	0.000	0.038	0.002	0.381	0.285	0.27
185	K	E	0.005	0.021	0.000	0.479	0.014	0.225	0.078	0.17
186	V	E	0.004	0.008	0.000	0.699	0.007	0.010	0.053	0.22
187	L	E	0.004	0.005	0.000	0.835	0.007	0.004	0.027	0.11
188	C	E	0.004	0.004	0.000	0.872	0.008	0.005	0.021	0.08
189	G	E	0.003	0.005	0.000	0.826	0.007	0.008	0.038	0.11
190	V	E	0.002	0.006	0.000	0.688	0.012	0.005	0.056	0.23
191	D	L	0.002	0.008	0.000	0.439	0.016	0.013	0.038	0.48
192	Y	L	0.003	0.010	0.000	0.086	0.007	0.067	0.114	0.71
193	S	L	0.002	0.008	0.000	0.010	0.006	0.094	0.186	0.69
194	H	L	0.021	0.014	0.000	0.003	0.008	0.074	0.345	0.53
195	D	L	0.185	0.026	0.000	0.003	0.009	0.084	0.192	0.50
196	K	H	0.636	0.035	0.000	0.001	0.001	0.166	0.084	0.07
197	R	H	0.804	0.025	0.000	0.001	0.001	0.106	0.024	0.03
198	R	H	0.947	0.013	0.000	0.001	0.001	0.016	0.009	0.01
199	E	H	0.984	0.004	0.000	0.000	0.000	0.005	0.002	0.00
200	R	H	0.993	0.002	0.000	0.000	0.000	0.003	0.001	0.00
201	A	H	0.996	0.001	0.000	0.000	0.000	0.002	0.000	0.00
202	V	H	0.997	0.000	0.000	0.000	0.000	0.002	0.000	0.00
203	A	H	0.997	0.000	0.000	0.000	0.000	0.001	0.000	0.00
204	I	H	0.996	0.000	0.000	0.000	0.000	0.002	0.000	0.00
205	V	H	0.996	0.000	0.000	0.000	0.000	0.002	0.000	0.00
206	R	H	0.996	0.000	0.000	0.000	0.000	0.002	0.000	0.00
207	L	H	0.997	0.000	0.000	0.000	0.000	0.001	0.000	0.00
208	V	H	0.994	0.001	0.000	0.000	0.000	0.003	0.000	0.00
209	L	H	0.988	0.002	0.000	0.001	0.000	0.006	0.001	0.00
210	G	H	0.889	0.006	0.000	0.000	0.000	0.101	0.003	0.00
211	F	H	0.574	0.006	0.001	0.000	0.000	0.393	0.010	0.01
212	L	H	0.961	0.004	0.001	0.000	0.001	0.020	0.004	0.01
213	W	H	0.994	0.001	0.000	0.000	0.000	0.002	0.001	0.00
214	P	H	0.991	0.001	0.000	0.000	0.000	0.006	0.000	0.00
215	L	H	0.996	0.001	0.000	0.000	0.000	0.002	0.000	0.00
216	L	H	0.995	0.001	0.000	0.000	0.000	0.002	0.000	0.00
217	T	H	0.995	0.001	0.000	0.000	0.000	0.003	0.000	0.00
218	L	H	0.995	0.001	0.000	0.000	0.000	0.003	0.000	0.00
219	T	H	0.995	0.001	0.000	0.000	0.000	0.003	0.000	0.00
220	I	H	0.995	0.000	0.000	0.000	0.000	0.002	0.000	0.00
221	C	H	0.995	0.000	0.000	0.000	0.000	0.002	0.000	0.00
222	Y	H	0.996	0.000	0.000	0.000	0.000	0.002	0.000	0.00
223	T	H	0.996	0.000	0.000	0.000	0.000	0.002	0.000	0.00
224	F	H	0.995	0.000	0.000	0.000	0.000	0.002	0.000	0.00
225	I	H	0.994	0.001	0.000	0.000	0.000	0.004	0.000	0.00
226	L	H	0.990	0.001	0.000	0.001	0.000	0.004	0.001	0.00
227	L	H	0.971	0.003	0.000	0.001	0.000	0.010	0.002	0.01
228	R	H	0.925	0.007	0.000	0.002	0.001	0.036	0.006	0.02
229	T	H	0.804	0.010	0.000	0.002	0.001	0.103	0.014	0.06
230	W	H	0.526	0.011	0.000	0.002	0.003	0.174	0.089	0.19
231	S	L	0.186	0.014	0.000	0.002	0.004	0.167	0.201	0.42
232	R	L	0.096	0.018	0.000	0.002	0.005	0.166	0.241	0.47
233	R	L	0.048	0.018	0.000	0.002	0.003	0.079	0.134	0.71
234	A	L	0.036	0.021	0.000	0.001	0.002	0.031	0.198	0.71
235	T	L	0.048	0.029	0.000	0.002	0.003	0.042	0.107	0.76
236	R	L	0.093	0.038	0.000	0.002	0.005	0.035	0.083	0.74
237	S	L	0.139	0.029	0.000	0.002	0.003	0.024	0.061	0.74
238	T	L	0.428	0.018	0.000	0.002	0.004	0.020	0.035	0.49
239	K	H	0.802	0.007	0.000	0.001	0.001	0.013	0.015	0.16
240	T	H	0.928	0.005	0.000	0.001	0.000	0.012	0.006	0.04
241	L	H	0.982	0.003	0.000	0.000	0.000	0.007	0.001	0.00
242	K	H	0.993	0.001	0.000	0.000	0.000	0.003	0.000	0.00
243	V	H	0.995	0.000	0.000	0.000	0.000	0.001	0.000	0.00
244	V	H	0.996	0.000	0.000	0.000	0.000	0.001	0.000	0.00
245	V	H	0.996	0.000	0.000	0.000	0.000	0.002	0.000	0.00
246	A	H	0.996	0.000	0.000	0.000	0.000	0.002	0.000	0.00
247	V	H	0.995	0.000	0.000	0.000	0.000	0.002	0.000	0.00
248	V	H	0.996	0.000	0.000	0.000	0.000	0.002	0.000	0.00
249	A	H	0.995	0.001	0.000	0.000	0.000	0.002	0.000	0.00
250	S	H	0.996	0.001	0.000	0.000	0.000	0.002	0.000	0.00
251	F	H	0.993	0.001	0.000	0.000	0.000	0.004	0.000	0.00
252	F	H	0.985	0.003	0.000	0.001	0.000	0.008	0.001	0.00
253	I	H	0.936	0.009	0.001	0.001	0.000	0.049	0.002	0.00
254	F	H	0.664	0.015	0.001	0.000	0.000	0.307	0.007	0.00
255	W	H	0.686	0.016	0.001	0.000	0.001	0.273	0.009	0.01
256	L	H	0.887	0.008	0.001	0.001	0.002	0.054	0.022	0.02
257	P	H	0.959	0.004	0.001	0.001	0.001	0.024	0.004	0.00
258	Y	H	0.986	0.002	0.000	0.000	0.000	0.007	0.001	0.00
259	Q	H	0.993	0.002	0.000	0.000	0.000	0.003	0.000	0.00
260	V	H	0.996	0.002	0.000	0.000	0.000	0.001	0.000	0.00
261	T	H	0.995	0.002	0.000	0.000	0.000	0.002	0.000	0.00
262	G	H	0.991	0.002	0.000	0.000	0.000	0.005	0.000	0.00
263	I	H	0.987	0.004	0.000	0.000	0.000	0.006	0.001	0.00
264	M	H	0.968	0.013	0.000	0.000	0.000	0.014	0.002	0.00
265	M	H	0.857	0.079	0.000	0.000	0.000	0.056	0.003	0.00
266	S	H	0.728	0.092	0.000	0.000	0.000	0.152	0.011	0.01
267	F	H	0.544	0.078	0.000	0.001	0.001	0.287	0.053	0.03
268	L	S	0.209	0.016	0.000	0.004	0.015	0.161	0.329	0.26
269	E	L	0.008	0.003	0.000	0.002	0.004	0.015	0.124	0.84
270	P	T	0.006	0.011	0.000	0.001	0.003	0.485	0.144	0.35
271	S	T	0.004	0.013	0.000	0.002	0.005	0.512	0.206	0.25
272	S	S	0.009	0.011	0.000	0.002	0.003	0.020	0.603	0.35
273	P	L	0.316	0.029	0.000	0.001	0.001	0.175	0.046	0.43
274	T	H	0.534	0.039	0.000	0.001	0.003	0.155	0.047	0.22
275	F	H	0.790	0.038	0.000	0.002	0.002	0.042	0.030	0.09
276	L	H	0.871	0.025	0.000	0.001	0.001	0.028	0.018	0.05
277	L	H	0.943	0.012	0.000	0.001	0.001	0.016	0.007	0.02
278	L	H	0.988	0.003	0.000	0.001	0.000	0.003	0.001	0.00
279	K	H	0.995	0.001	0.000	0.000	0.000	0.002	0.000	0.00
280	K	H	0.995	0.001	0.000	0.000	0.000	0.003	0.000	0.00
281	L	H	0.996	0.000	0.000	0.000	0.000	0.002	0.000	0.00
282	D	H	0.998	0.000	0.000	0.000	0.000	0.001	0.000	0.00
283	S	H	0.997	0.000	0.000	0.000	0.000	0.001	0.000	0.00
284	L	H	0.997	0.000	0.000	0.000	0.000	0.002	0.000	0.00
285	C	H	0.997	0.001	0.000	0.000	0.000	0.002	0.000	0.00
286	V	H	0.995	0.001	0.000	0.000	0.000	0.003	0.000	0.00
287	S	H	0.989	0.003	0.000	0.000	0.000	0.007	0.000	0.00
288	F	H	0.975	0.006	0.000	0.000	0.000	0.017	0.001	0.00
289	A	H	0.942	0.008	0.001	0.000	0.000	0.044	0.002	0.00
290	Y	H	0.845	0.010	0.001	0.000	0.000	0.126	0.010	0.00
291	I	H	0.759	0.018	0.001	0.001	0.003	0.167	0.028	0.02
292	N	H	0.670	0.057	0.001	0.001	0.002	0.161	0.033	0.07
293	C	H	0.500	0.092	0.001	0.000	0.001	0.380	0.015	0.01
294	C	H	0.439	0.081	0.000	0.000	0.000	0.437	0.017	0.02
295	I	H	0.846	0.030	0.001	0.001	0.004	0.075	0.022	0.02
296	N	H	0.904	0.003	0.000	0.001	0.001	0.009	0.008	0.07
297	P	H	0.993	0.002	0.000	0.000	0.000	0.002	0.000	0.00
298	I	H	0.997	0.001	0.000	0.000	0.000	0.001	0.000	0.00
299	I	H	0.995	0.001	0.000	0.000	0.000	0.002	0.000	0.00
300	Y	H	0.994	0.002	0.000	0.000	0.000	0.003	0.000	0.00
301	V	H	0.983	0.003	0.000	0.000	0.000	0.012	0.001	0.00
302	V	H	0.972	0.003	0.000	0.000	0.000	0.021	0.002	0.00
303	A	H	0.850	0.003	0.000	0.001	0.001	0.091	0.048	0.00
304	G	L	0.028	0.001	0.000	0.001	0.002	0.007	0.433	0.52
305	Q	H	0.915	0.014	0.000	0.000	0.000	0.038	0.023	0.01
306	G	H	0.933	0.012	0.000	0.000	0.000	0.030	0.012	0.01
307	F	H	0.964	0.014	0.000	0.001	0.000	0.010	0.004	0.00
308	Q	H	0.989	0.003	0.000	0.000	0.000	0.003	0.001	0.00
309	G	H	0.992	0.002	0.000	0.000	0.000	0.003	0.001	0.00
310	R	H	0.993	0.001	0.000	0.000	0.000	0.002	0.000	0.00
311	L	H	0.996	0.001	0.000	0.000	0.000	0.002	0.000	0.00
312	R	H	0.992	0.001	0.000	0.000	0.000	0.004	0.000	0.00
313	K	H	0.971	0.002	0.000	0.001	0.000	0.021	0.001	0.00
314	S	H	0.936	0.004	0.000	0.001	0.000	0.049	0.003	0.00
315	L	H	0.763	0.020	0.000	0.004	0.002	0.052	0.050	0.10
316	P	H	0.732	0.054	0.000	0.011	0.003	0.043	0.015	0.14
317	S	H	0.724	0.062	0.000	0.023	0.004	0.046	0.021	0.11
318	L	H	0.688	0.054	0.000	0.036	0.006	0.049	0.025	0.14
319	L	H	0.570	0.028	0.000	0.042	0.006	0.058	0.035	0.26
320	R	H	0.428	0.026	0.000	0.041	0.008	0.125	0.054	0.31
321	N	L	0.240	0.028	0.000	0.027	0.004	0.157	0.038	0.50
322	V	L	0.153	0.026	0.000	0.014	0.005	0.063	0.037	0.70
323	L	L	0.051	0.010	0.000	0.005	0.002	0.012	0.027	0.89
324	T	L	0.033	0.008	0.000	0.005	0.001	0.006	0.012	0.93
325	E	L	0.035	0.010	0.000	0.009	0.002	0.013	0.013	0.91
326	E	L	0.032	0.011	0.000	0.012	0.002	0.013	0.011	0.91
327	S	L	0.025	0.007	0.000	0.018	0.002	0.006	0.008	0.93
328	V	L	0.021	0.004	0.000	0.036	0.004	0.005	0.009	0.92
329	V	L	0.009	0.002	0.000	0.022	0.002	0.003	0.006	0.95
330	R	L	0.003	0.002	0.000	0.006	0.001	0.003	0.005	0.98
331	E	L	0.003	0.002	0.000	0.003	0.001	0.004	0.006	0.98
332	S	L	0.003	0.002	0.000	0.001	0.000	0.003	0.004	0.98
333	K	L	0.005	0.002	0.000	0.002	0.001	0.004	0.006	0.98
334	S	L	0.005	0.002	0.000	0.002	0.001	0.004	0.004	0.98
335	F	L	0.008	0.003	0.000	0.002	0.001	0.006	0.008	0.97
336	T	L	0.007	0.003	0.000	0.002	0.001	0.009	0.010	0.96
337	R	L	0.013	0.003	0.000	0.002	0.001	0.007	0.009	0.96
338	S	L	0.023	0.003	0.000	0.001	0.000	0.010	0.010	0.95
339	T	L	0.083	0.002	0.000	0.004	0.001	0.013	0.014	0.88
340	V	L	0.322	0.004	0.000	0.009	0.003	0.008	0.009	0.64
341	D	L	0.350	0.004	0.000	0.003	0.000	0.008	0.007	0.62
342	T	H	0.597	0.009	0.000	0.003	0.001	0.019	0.008	0.36
343	M	H	0.649	0.006	0.000	0.003	0.001	0.017	0.009	0.31
344	A	H	0.686	0.005	0.000	0.002	0.000	0.006	0.006	0.29
345	Q	H	0.815	0.003	0.000	0.003	0.000	0.006	0.005	0.16
346	K	H	0.828	0.003	0.000	0.005	0.001	0.007	0.004	0.15
347	T	H	0.694	0.003	0.000	0.004	0.001	0.013	0.004	0.28
348	Q	L	0.113	0.003	0.000	0.002	0.001	0.028	0.017	0.83
349	A	L	0.014	0.002	0.000	0.001	0.001	0.013	0.000	0.96
350	V	L	0.000	0.000	0.000	0.000	0.000	0.000	0.000	0.99
