# Supplementary figures and images for: Helix 8 in chemotactic receptors of the complement system
Source: PLoS Comput Biol. 2022 Jul 21;18(7):e1009994. doi: 10.1371/journal.pcbi.1009994 (PMC9359563; doi:10.1371/journal.pcbi.1009994)

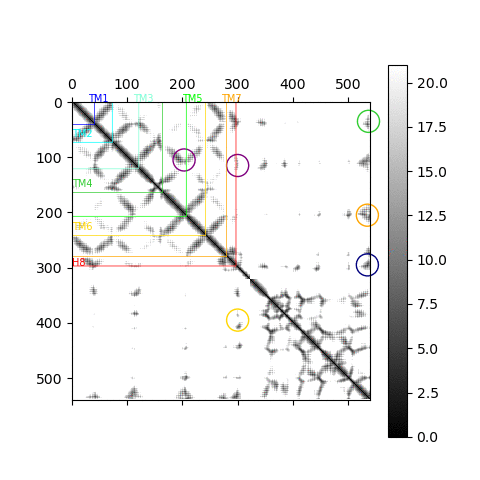

Supplement: S2 Appendix — Distances were computed between Cα atoms of the receptor and the Gα subunit from every tenth ns of the 1.5 μ MD simulation started from the active conformation of C5aR1 based on FPR2. (GIF) [file pcbi.1009994.s002.gif]

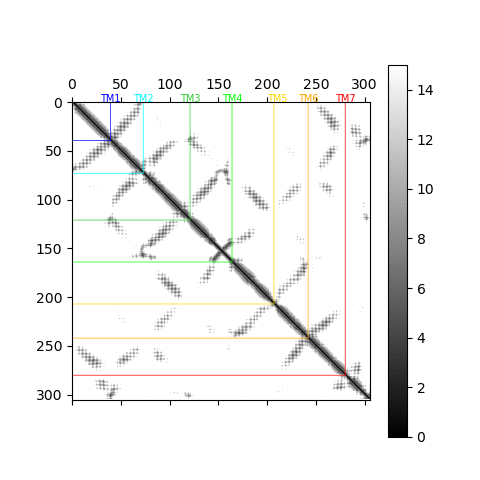

Supplement: S3 Appendix — Distances were computed between Cα atoms of the receptor from every tenth ns of the 1.5 μ MD simulation started from the crystal structure of the inactive conformation of C5aR1. (GIF) [file pcbi.1009994.s003.gif]
